# Zinc silicate modulates bone substitute degradation following macrophage activation via the JAK/STAT pathway and expedites the initiation of bone repair: *in vitro* and *in vivo* studies

**DOI:** 10.1093/rb/rbag037

**Published:** 2026-03-05

**Authors:** Jingdi Chen, Xiaotian Hao, Chunxing Xian, Xiang He, Taoran Wang, Jiakai Gao, Tao Liu, Wei Wu, Zhao Yang, Long Bi

**Affiliations:** Department of Orthopedics, Xijing Hospital, Air Force Medical University, Changle West Street, Xi'an, Shaanxi 710032, China; Department of Orthopedics, 95829 Military Hospital in PLA, Gongnongbing Road, Jiang'an District, Wuhan, Hubei 430000, China; Department of Orthopedics, Xijing Hospital, Air Force Medical University, Changle West Street, Xi'an, Shaanxi 710032, China; Department of Orthopedics, Xijing Hospital, Air Force Medical University, Changle West Street, Xi'an, Shaanxi 710032, China; Department of Orthopedics, Xijing Hospital, Air Force Medical University, Changle West Street, Xi'an, Shaanxi 710032, China; Department of Orthopedics, Xijing Hospital, Air Force Medical University, Changle West Street, Xi'an, Shaanxi 710032, China; Department of Orthopedics, Xijing Hospital, Air Force Medical University, Changle West Street, Xi'an, Shaanxi 710032, China; Department of Orthopedics, 95829 Military Hospital in PLA, Gongnongbing Road, Jiang'an District, Wuhan, Hubei 430000, China; Department of Critical Care Medicine, Renmin Hospital of Wuhan University, Zhangzhidong Road, Wuchang District, Wuhan, Hubei 430000, China; Department of Orthopedics, Xijing Hospital, Air Force Medical University, Changle West Street, Xi'an, Shaanxi 710032, China; Ankle Trauma and Degeneration Ward, Honghui Hospital, Xi’an Jiaotong University, Nanguo Road, Nanshaomen, Beilin District, Xi'an, Shaanxi 710054, China; Department of Orthopedics, Xijing Hospital, Air Force Medical University, Changle West Street, Xi'an, Shaanxi 710032, China

**Keywords:** macrophage, zinc silicate, osteogenesis, angiogenesis, degradable composites, JAK/STAT pathway

## Abstract

Repairing segmental bone defects remains difficult because conventional bone substitutes rarely integrate immunomodulation with efficient tissue regeneration. To address this limitation, zinc silicate/collagen/hydroxyapatite (ZCH) composites were developed wherein the incorporation of zinc allows controlled material degradation and imparts immunoreactive properties. The addition of moderate concentrations of zinc silicate (<0.337 M) maintained good biocompatibility while enabling tuneable degradation rates and bioactivity. Among the composites, the composite containing 0.224 M zinc silicate (10ZCH) exhibited the most favourable balance between material degradation and bone regeneration and promoted accelerated angiogenesis and osteogenesis. Mechanistic studies revealed that 10ZCH transiently activated M1 macrophages and promoted their polarization towards the proregenerative M2 phenotype by activating the JAK/STAT pathway. These findings demonstrate that rational zinc incorporation can orchestrate immune responses, improve material resorption and promote rapid tissue repair. This work provides an innovative design concept for fabricating immune-responsive and resorbable bone graft substitutes that couple degradation dynamics with regenerative efficacy.

## Graphical abstract

**Graphical Abstract:**
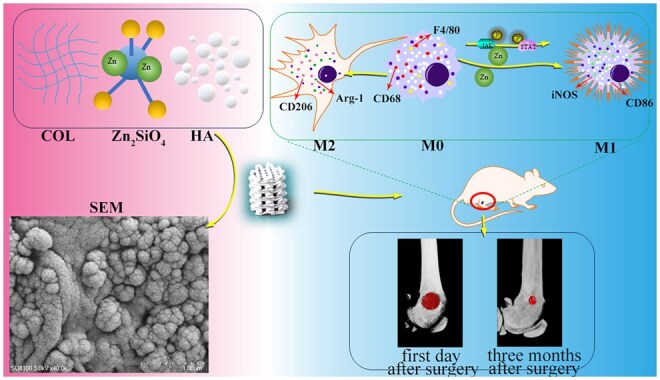

## Introduction

Segmental bone defects caused by infection, trauma or congenital anomalies remain a major clinical challenge because achieving both mechanical stability and biological regeneration is difficult [1, 2]. Approximately 20 million individuals are affected by bone defects each year worldwide, nearly 60% of whom require bone grafting procedures [3]. Autologous bone grafting is considered the gold standard because of its osteoconductive, osteoinductive and osteogenic potential, but its clinical application is hindered by donor site morbidity and limited graft availability [4]. Allografts offer an alternative tissue source and may induce beneficial immunological stimuli to promote bone formation; however, safety concerns such as immune rejection and disease transmission remain [1].

To overcome these limitations, synthetic bone substitute materials have attracted significant attention. As essential components for bone tissue engineering, ideal bone substitutes should combine biocompatibility, biodegradability, osteoconductivity and mechanical strength with an appropriate microarchitecture [2, 5]. Compared with nondegradable materials, degradable bone substitutes allow for gradual resorption and progressive stress transfer to the regenerating tissue, thereby facilitating osseointegration and functional recovery [1, 6]. However, it is difficult to fabricate biodegradable materials with a degradation rate that precisely matches that of the dynamic process of new bone formation, which remains a key bottleneck for clinical translation [1].

Recently, bone immunology studies have revealed that the success of bone repair depends not only on the intrinsic osteogenic potential of the materials but also on their ability to orchestrate the host immune response at the implantation site [1, 7]. This concept, termed osteoimmunomodulation, highlights the importance of balancing the proinflammatory and anti-inflammatory phases for optimal bone regeneration [8, 9]. Upon implantation, biomaterials interact with immune cells, such as macrophages, triggering the release of cytokines that govern angiogenesis, osteogenesis and even material degradation. As key mediators of innate immunity, macrophages exhibit remarkable plasticity and can switch between proinflammatory (M1) and proregenerative (M2) phenotypes in response to local stimuli [10–12]. Therefore, regulating macrophage polarization through rational material design has emerged as a promising strategy to improve bone repair outcomes [13–15], effectively serving as a functional bridge between the immune system and skeletal system.

Metallic elements with immunoregulatory potential have attracted increasing attention for bone tissue engineering applications [16]. Zinc, the second most abundant trace element after iron [17], is involved in nucleic acid metabolism, signal transduction and enzyme activation [18, 19]. Zinc modulates the activity of osteoblasts and osteoclasts, promoting osteogenesis while inhibiting bone resorption [7, 20, 21]. In addition, zinc plays a crucial role in immune regulation [22, 23], and its moderate degradation rate, compared with those of magnesium and iron, makes it particularly suitable for use in biodegradable materials [24].

Our previous studies demonstrated that zinc silicate promotes macrophage polarization towards the M2 phenotype, reduces inflammation and infection [25, 26] and enhances angiogenesis and osteogenesis through activation of the p38 MAPK pathway [27]. However, whether zinc silicate can regulate material degradation through macrophage-mediated mechanisms, which are critical for achieving coordinated immune and degradation responses during bone healing, remains unclear.

Previous studies have explored the application of biodegradable materials in the management of bone infections, bone defects and diabetic wound healing [28–31]. However, current research focuses predominantly on the bone repair functions of biomaterials, such as their antimicrobial activity, promotion of vascularization and neurogenic or osteogenic properties, while the degradation mechanisms have received considerably less attention [32]. A systematic strategy that concurrently addresses the modulation of inflammation, infection control, osteogenesis, angiogenesis and tuneable material degradation aligned with tissue regeneration dynamics remains largely unexplored.

In this study, we developed zinc silicate/collagen/hydroxyapatite (ZCH) composites designed to exhibit both immunoregulation and controllable degradation. We systematically investigated their effects on macrophage polarization, angiogenesis and osteogenesis and examined their degradation kinetics, aiming to elucidate the mechanism underlying zinc-mediated osteoimmune regulation and its impact on osseointegration.

**Figure 1:**
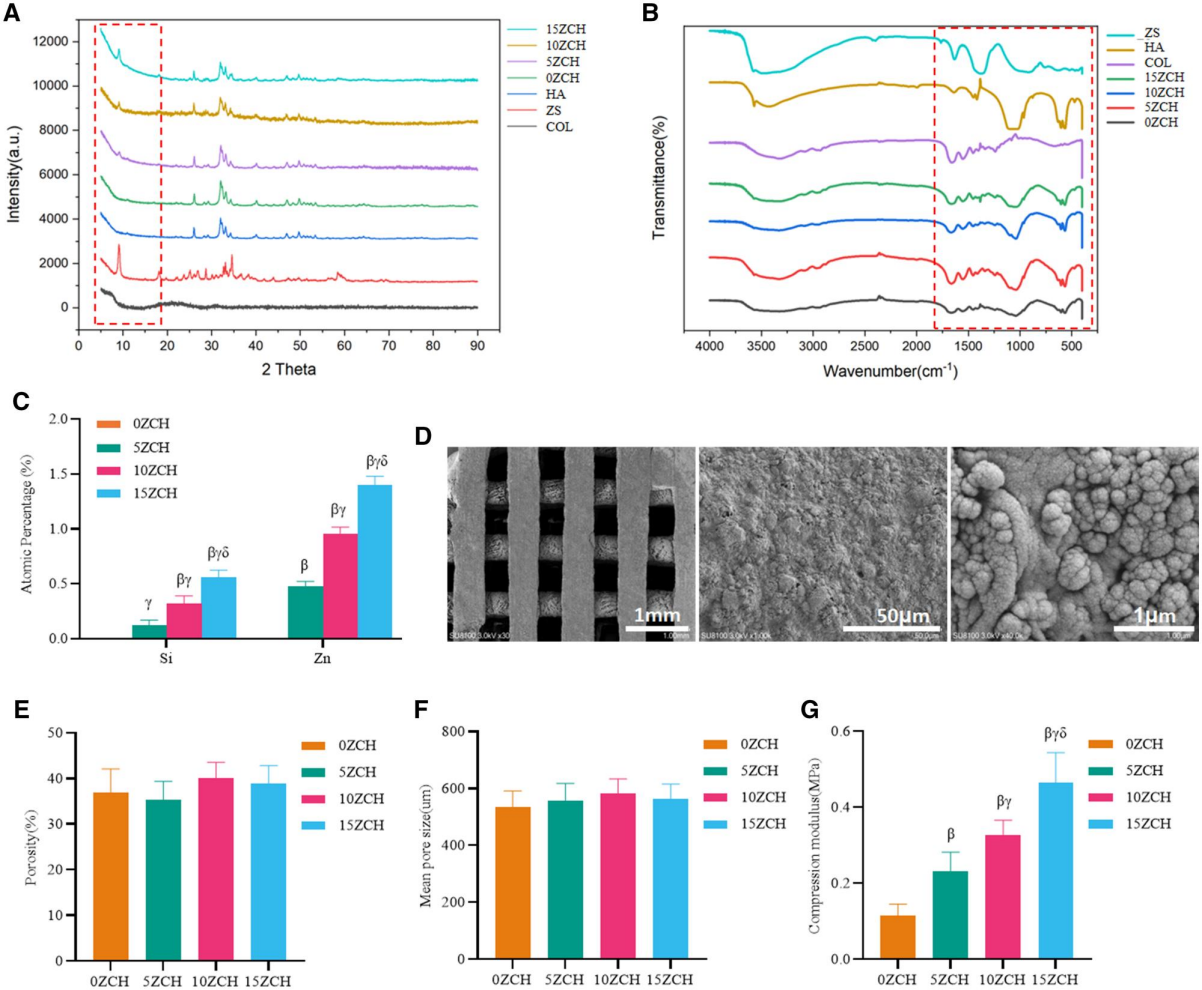
Physical characteristics of the composites. (**A**) shows the XRD results (The characteristic peaks of hydroxyapatite are primarily located at 2θ = 25.8°, 31.7°, 32.2°, and 32.8°, the characteristic peaks of zinc silicate are mainly observed at 2θ = 10°, 32°, and 35°.), (**B**) shows the Fourier infrared spectra (Collagen exhibits absorption peaks at 3600 cm^−^¹–3200 cm^−^¹, 1630 cm^−^¹, 1550 cm^−^¹, and 1250 cm^−^¹,  Hydroxyapatite shows characteristic absorption peaks at 550 cm^−^¹, 1050 cm^−^¹, and 3530 cm^−^¹. Zinc silicate displays characteristic absorption peaks at 1640 cm^−^¹ and 1350 cm^−^¹), (**C**) shows the composition ratios of silicon and zinc in the composites with different concentrations determined by energy spectrum analysis and (**D**) shows scanning electron microscopy images of the composites (1.0 K and 40.0 K). (**E**) shows the average porosities of the composite scaffolds. (**F**) displays the average pore sizes of the composite scaffolds. (**G**) presents the compressive moduli of the composite scaffolds. ZS: zinc silicate; HA: hydroxyapatite; COL: collagen. *β* represents *P* < 0.05 compared with the 0ZCH group, *γ* represents *P* < 0.05 compared with the 5ZCH group, and *δ* represents *P* < 0.05 compared with the 10ZCH group.

## Materials and methods

### Material preparation and characterization

#### Synthesis of zinc silicate via a hydrothermal method

The synthesis of zinc silicate was carried out using a hydrothermal method, as previously described [27, 33]. Briefly, zinc nitrate hexahydrate [Zn(NO_3_)_2_⋅6H_2_O] (Sinopharm Chemical Reagent Co., Ltd, China) and tetraethyl orthosilicate (TEOS; Aladdin, China) were mixed in distilled water at a molar ratio of 2:1. A 25-wt% aqueous ammonia solution (McLean, China) was subsequently added to adjust the pH to 7.0. The reaction mixture was stirred thoroughly and then transferred to a hydrothermal reactor (CEM Corporation, USA), where it was heated at 170°C for 6 h. The resulting product was washed three times with deionized water and anhydrous ethanol and freeze-dried at −80°C for 24 h.

#### Collagen extraction and identification

Commercial porcine skin (Aperture Biotechnology Co., Ltd, China) was used as the collagen source. Subcutaneous fat was mechanically removed from the skin until it became translucent. The tissue was then defatted using a chloroform: methanol mixture (1:1 v/v; both from Aladdin, China), followed by enzymatic digestion with pepsin (Solebaol, China). After digestion, the mixture was centrifuged at 18 000 rpm for 10 minutes at 4°C. The resulting supernatant was collected and transferred to dialysis tubing (molecular weight cut-off: 8000–14 000 Da; Solebaol, China) and dialyzed against deionized water for 3 days, during which time the water was changed every 6 h. After dialysis, the collagen solution was freeze-dried for further use.

To confirm that the extracted substance was primarily type I collagen, liquid chromatography–mass spectrometry (LC–MS) analysis was performed [34]. Briefly, 9.8 mg of freeze-dried collagen was dissolved in deionized water, followed by the sequential addition of ammonium bicarbonate (NH_4_HCO_3_; Sigma, USA) and trypsin (Promega, USA) for enzymatic digestion. The resulting peptide mixture was desalted, and the supernatant was collected for LC–MS analysis (Thermo Fisher Scientific, USA).

#### Construction of 3D-printed ZCH composites

Two grams of collagen were dissolved in 8 mL of 0.5 M acetic acid (Jinshan Chemical Reagent, China) to form a gel-like solution. Once the collagen was fully dissolved, 4 g of hydroxyapatite powder (Nanjing Aprui Nanomaterials Co., Ltd, China) was added and the mixture was stirred thoroughly until homogeneous. The resulting paste was loaded into the extruder of a 3D bioprinter and degassed to remove air bubbles.

Similarly, 3D-printed inks containing ZCH composites with final zinc silicate concentrations of 0.112, 0.224 and 0.337 M were prepared according to the formulations listed in Table 1. These composites were designated 5ZCH, 10ZCH and 15ZCH, respectively.

**Table 1:** Composition ratios of ZCH composites containing different concentrations of zinc silicate.

	Collagen （g）	Zinc silicate （g）	Hydroxyapatite （g）
0ZCH	2.0	0.0	4.0
5ZCH	2.0	0.2	3.8
10ZCH	2.0	0.4	3.6
15ZCH	2.0	0.6	3.4

Note: 0ZCH: ZCH composites containing 0 M zinc ion concentration; 5ZCH: ZCH composites containing 0.112 M zinc silicate concentration; 10ZCH: ZCH composites containing 0.224 M zinc silicate concentration; 15ZCH: ZCH composites containing 0.337 M zinc silicate concentration.

#### Preprinting model design and fabrication

The preprinted model was designed using a 3D printer (Xi’an Dianyun Biotechnology Co., Ltd, China). The filaments were fabricated into a 10 × 10 × 2 mm cube and a cylinder with a diameter of 3.5 mm and height of 5 mm; the nozzle diameter was set to 0.4 mm. The printing parameters were as follows: a printing speed of 6 mm/s, infill density of 40% and extrusion width of 0.4 mm, and each layer was printed twice to ensure adequate porosity of the composites. All printing was conducted at room temperature to preserve the bioactivity of the collagen. Immediately after printing, the mixture was stored at −80°C for 1 h and then freeze-dried using a vacuum lyophilizer (Ningbo SCIENTZ Biotechnology Co., Ltd, China).

The freeze-dried composites were immersed in a 1% Genipin crosslinking solution (Solepol, China) at 4°C for 24 h. After crosslinking, the composites were rinsed three times, freeze-dried again and sealed in sterilization indicator bags (8465A; Aoquan Medical Technology Co., Ltd, China). Finally, following the same sterilization protocol used for clinical implants, the composites were sterilized by ethylene oxide treatment (600 mg/L ethylene oxide at 55°C with 60% relative humidity) for 6 h and stored aseptically for subsequent experiments.

#### Physicochemical characterization of the composites

The composites containing different concentrations of zinc silicate were ground into a fine powder. Zinc silicate powder, hydroxyapatite powder (EMPEROR NANO, China) and collagen were used as reference materials. X-ray diffraction (XRD; Philips, The Netherlands) and Fourier transform infrared (FTIR) spectroscopy (Hitachi, Ltd, Japan) were employed to analyse the compositions of the composites. To increase the conductivity of the composite surfaces, the samples were sputter-coated with carbon. The elemental composition and atomic percentages of the composites were then determined using inductively coupled plasma–optical emission spectrometry (ICP–OES; Thermo Fisher Scientific, USA). The 3D composite materials were mounted on carbon stickers, placed on a Lon Sputtering apparatus (Hitachi Corporation, Japan) and sputter-coated with gold for 30 s. The coated samples were subsequently observed by scanning electron microscopy (SEM; Hitachi Corporation, Japan).

The average porosity and pore size of each group were evaluated using micro-CT. Samples measuring 10 × 10 × 2 mm were scanned at 80 kV and 5 W. Porosity was calculated using VGStudio 2.1 software (*n* = 3). The material density within the samples was positively correlated with the pixel values obtained from the micro-CT 3D reconstructed images. Since the scaffold pores, which contained air (lower density), corresponded to lower pixel values, the air volume within the region of interest was determined by analysing the distribution range of the air pixel values. The average porosity was calculated as the ratio of the air volume to the total scaffold volume. The average pore size was defined as the mean diameter of the largest sphere that could fit within the pores.

Samples of all four types of scaffolds (each 10 × 10 × 2 mm) were immersed in simulated body fluid (SBF) at 37°C for 24 h to mimic the postimplantation environment. Compression testing was subsequently performed using a Model 858 materials testing system at a compression rate of 0.083 mm/s and a 1-N preload. The compressive modulus was calculated as the slope of the stress–strain curve within the elastic deformation region.

### *In vitro* study of the interaction between composites and macrophages

#### Macrophage recovery and subculture

One millilitre of thawed macrophage suspension (Pricella Biotechnology, China) was mixed with 9 mL of complete medium, consisting of 45 mL of Dulbecco’s modified Eagle’s medium (DMEM; HyClone, USA), 10 mL of foetal bovine serum (FBS; Sigma–Aldrich, St. Louis, USA) and 500 μL of 1% v/v streptomycin solution (Sigma–Aldrich, St. Louis, USA). After being washed and centrifuged, the pellet was retained. One millilitre of the cell suspension (at a density of ∼10^5^ cells/mL) was then added to 9 mL of complete medium and incubated for 24 h before being subcultured under standard conditions.

#### Effects of the composites on macrophage morphology

After being soaked in cell culture medium, the cubic scaffolds 0ZCH, 5ZCH, 10ZCH and 15ZCH were placed in a 12-well plate (*n* = 3 per group). Each scaffold was then seeded with 100 μL of macrophage suspension at a uniform cell density, after which 2 mL of fresh culture medium was added. The plate was subsequently transferred to an incubator for coculture. After 1, 3 and 7 days of incubation, the macrophages were fixed with 2.5% glutaraldehyde, dehydrated, dried under vacuum and sputter-coated with gold. The morphological changes in the macrophages were observed using scanning electron microscopy and live/dead staining was performed after 1, 2 and 3 days of coculture.

Additionally, total RNA was extracted from the cocultured macrophages on Days 3 and 7 using an RNA extraction kit (TaKaRa, China) following the manufacturer’s instructions. After the RNA was quantified, 1 μL of total RNA was reverse transcribed with an RNA reverse transcription kit (TaKaRa, China) to generate cDNA. Quantitative real-time polymerase chain reaction (qRT–PCR) was performed using a qRT–PCR system (Bio-Rad Laboratories, Inc., USA) with TB Green Premix Ex Taq II (TaKaRa, China). GAPDH was used as a reference gene to normalize the expression levels of the target genes. The primers used in this experiment are listed in Table 2.

**Table 2:** Real-time polymerase chain reaction primers.

Gene	5′-3′ Primer (Forwards)	3′-5′ Primer (Reverse)
F4/80	CgCCAggTACgAgATgAATATAg	CgTCTTAgAAgTggAAggCATAg
CD68	CTCTTgCTgCCTCTCATCATT	CTggTAggTTgATTgTCgTCTC
CD86	ggATACCCgAAACCTACAAAgA	CATCCgggAATggAAgAgATAg
iNOS	CCTggAggTTCTAgATgAgAgT	TAgTgATgTCCAggAAgTAggT
Arg1	TCATggAAgTgAACCCAACTC	AggTAgTCAgTCTCTggCTTAT
CD206	gCAggTggTTTATgggATgT	TgggTTCAggAgTTgTTgTg
GAPDH	ACAgCAACAgggTggTggAC	TTTgAgggTgCAgCgAACTT

#### Effect of macrophages on zinc-loaded composite degradation

0ZCH, 5ZCH, 10ZCH and 15ZCH composite cubes were freeze-dried and weighed, with the initial weight recorded as W_0_. After the composites were dissolved in cell culture medium, they were placed in 12-well plates in triplicate. A total of 100 μL of macrophage suspension, at equal concentrations, was added to each well, followed by the addition of 2 mL of complete culture medium. The samples were then cocultured in an incubator. Composite degradation was monitored at 1, 3, 7, 14, 21 and 28 days. After each time point, the samples were freeze-dried and reweighed, with the corresponding weights recorded as W_1_, W_3_, W_7_, W_14_, W_21_ and W_28_. Volume changes were assessed using microcomputed tomography (micro-CT). Every 2 days, the culture medium was replaced and the absorbance of the previous media was measured at 450 nm to evaluate the extent of composite degradation.

### Analyses of composite material safety *in vivo*

#### Animal experiments

All animal experiments were performed in accordance with the *Guidelines for the Care and Use of Laboratory Animals* published by the National Academy of Sciences of the United States (NAS) and approved by the Welfare and Ethics Committee of the Laboratory Animal Centre of our university (approval number: IACUC-20230121), and are reported following the ARRIVE guidelines [35]. A total of 90 10-week-old male Sprague–Dawley rats weighing ∼300 g were purchased from the Experimental Animal Centre of our university. The rats were randomly assigned to the following groups on the basis of body weight and length so that there were no significant differences among the groups: the control group, 0ZCH group, 5ZCH group, 10ZCH group and 15ZCH group.

The Sprague–Dawley rats were anaesthetized via intraperitoneal injection of 1% pentobarbital sodium (40 mg/kg). The lateral condyle of the right femur was selected as the target region [36]. After routine skin preparation and sterilization, a longitudinal incision of ∼1 cm was made. The skin and subcutaneous tissue were carefully dissected, and the muscle was bluntly stripped away from the femoral condyle. A bone defect measuring 3 mm in diameter with a depth of 5 mm was created at the proximal epiphyseal line of the femoral condyle and perpendicular to the femoral shaft.

In accordance with the experimental design, the rats in the control group did not receive an implant, whereas the other groups received the corresponding cylindrical material. The subcutaneous tissue and skin were then sutured, and the wound was wrapped and fixed after sterilization. To prevent infection, gentamicin sulphate (0.1 mL; 80 000 units/2 mL) was administered intraperitoneally immediately after surgery and on the first and second postoperative days.

#### Routine blood and biochemical tests

Two weeks after surgery, three Sprague–Dawley rats from each group were randomly selected. After anaesthesia, blood was collected from the venous plexus of the inner canthus, and the prothrombin time (PT), activated partial thromboplastin time (APTT), thrombin time (TT), levels of fibrinogen (Fib), alanine aminotransferase (ALT), aspartate aminotransferase (AST), alkaline phosphatase (ALP), urea (UA), creatinine (CR), creatine kinase isoenzyme (CK-MB), lactate dehydrogenase (LDH) and zinc ion concentration were measured using an automatic animal blood cell analyser (BC-5000vet, Shenzhen Mindray Animal Medical Co., Ltd) and an automatic biochemical analyser (Chemray, Shenzhen Laidu Life Technology Co., Ltd).

#### Determination of the zinc ion concentration in the tissue surrounding the bone defect

At 4 weeks after surgery, the bone tissue surrounding the defect site was excised and subjected to digestion with nitric acid. The zinc ion concentration was then determined using ICP-OES (ICP-OES7200, Thermo Fisher Scientific, USA).

#### Haematoxylin and eosin (H&E) staining

Four weeks after surgery, the Sprague–Dawley rats were euthanized by cervical dislocation following anaesthesia and were immediately injected with 4% paraformaldehyde (Biosharp, China). The heart, liver, spleen, lungs and kidneys were excised and fixed in 4% paraformaldehyde at 4°C for 24 h. After being embedded in paraffin, the tissue sections were cut into 5-µm-thick slices using a paraffin microtome (Leica Biosystems, China). The sections were then dewaxed with xylene, stained with haematoxylin and eosin (H&E) and examined under a light microscope (Carl Zeiss, Germany). For physicochemical assessment, changes in the pH and zeta potential at the material–tissue interface were monitored 4 weeks after surgery.

### *In vivo* analysis of the interaction between the composite materials and macrophages

#### qRT–PCR analysis

qRT–PCR analysis was performed to assess the gene expression of F4/80, CD68, CD86, iNOS, Arg1 and CD206. Two and four weeks after surgery, total RNA was extracted from the bone tissue surrounding the defect using an RNA extraction kit (TaKaRa, China) following the manufacturer’s instructions. Gene expression levels were then quantified as described in Effects of the composites on macrophage morphology.

#### Western blotting

The protein expression levels of F4/80, iNOS and Arg1 were assessed by western blotting. Two weeks after surgery, the bone tissue surrounding the defect was excised, washed with precooled PBS and incubated in RIPA lysis buffer (Servicebio, China) containing phenylmethyl sulfonyl fluoride (PMSF; Servicebio, China), a protease inhibitor cocktail (Servicebio, China) and a phosphatase inhibitor cocktail (Servicebio, China). The tissue was then homogenized using a high-speed, low-temperature tissue grinder (Servicebio, China), after which the supernatant was collected. The total protein concentration in the supernatant was quantified using a BCA protein assay kit (Solarbio, China), followed by electrophoresis, membrane transfer and blocking. The membrane was incubated overnight at 4°C with the primary antibody, followed by incubation with the secondary antibody at room temperature for 1 h. The protein bands were detected using an enhanced chemiluminescence detection kit (Servicebio, China) and visualized with an imaging system (version 5.1; Bio-Rad, USA). Quantitative analysis was performed using ImageJ software (version 1.80; National Institutes of Health, USA). β-Actin was used as the internal control.

The primary antibodies used in this experiment were anti-F4/80 (A18637; 1:2500; ABclonal), anti-iNOS (ab49999; 1:2500; Abcam), anti-Arg1 (16001-1-AP; 1:25000; Proteintech) and anti-β-actin (GB15003; 1:1500; Servicebio). The corresponding secondary antibodies were HRP-conjugated goat anti-rabbit IgG (H + L) (GB23303, 1:15000; Servicebio) and HRP-conjugated goat anti-rat IgG (H + L) (GB23302, 1:15000; Servicebio).

#### Immunohistochemistry and immunofluorescence

Two weeks after surgery, the bone tissue surrounding the defect was excised, washed with precooled PBS, fixed with 4% paraformaldehyde for 24 h, decalcified using EDTA decalcification solution and then embedded in paraffin.

The protein expression levels of CD68, CD86 and Arg1 in the bone tissue were assessed by immunofluorescence. After the samples were dewaxed and blocked with BSA, the primary antibody was added and the samples were incubated at 4°C overnight. The following day, the corresponding fluorescently labelled secondary antibody was added for incubation at room temperature. Heat treatment was applied, and other primary and secondary antibodies were added and incubated in a similar manner. The nuclei were then visualized under a fluorescence microscope (Carl Zeiss, Germany), and the fluorescence intensity was analysed using ImageJ software. The primary antibodies used were against CD68 (GB113109; Servicebio), CD86 (GB13585; Servicebio) and Arg1 (GB11285; Servicebio).

The protein expression levels of F4/80, iNOS and CD206 in the bone tissue around the defect were evaluated by immunohistochemistry. After dewaxing, the sections were blocked with BSA and incubated with primary antibodies against F4/80 (GB113373, Servicebio), iNOS (GB11119, Servicebio) or CD206 (GB113497, Servicebio) at 4°C overnight. The sections were then incubated with HRP-conjugated goat anti-rabbit IgG (GB23303; Servicebio) at room temperature for 1 h. After colour development using 3,3'-diaminobenzidine (DAB) solution (G1212; Servicebio), the sections were observed under a microscope and analysed using ImageJ software.

### Analysis of the degradation of the composite materials *in vivo*

micro-CT was used to assess the degradation of the ZCH composites at 4, 8 and 12 weeks after surgery. On the first day following surgery, three Sprague–Dawley rats were randomly selected from each group (0ZCH, 5ZCH, 10ZCH and 15ZCH). *In vivo* scanning was performed using micro-CT (Bruker, USA) under anaesthesia (layer thickness: 30 µm). The rats were then individually housed in five separate cages. Image reconstruction was carried out using the NRecon system, image display was performed with the CTVox system and region of interest analysis was conducted using the CTAn system. The bone tissue volume (BV_0_) and the volume of the region of interest (TV_0_) were calculated. The volume of the remaining undegraded composite material (VZCH_0_) was calculated as VZCH_0_ = TV_0_−BV_0_.

At 4, 8 and 12 weeks after surgery, the volumes of the remaining undegraded composites (VZCH_4_, VZCH_8_ and VZCH_12_) were measured using the same method. The degradation volume of each composite during the first month after surgery was calculated using the formula VZCH_1_ = VZCH_0_−VZCH_4_, the degradation volume after 2 months was calculated as VZCH_2_ = VZCH_0_−VZCH_8_ and the degradation volume after 3 months was calculated as VZCH_3_ = VZCH_0_−VZCH_12_.

### Evaluation of the osteogenic potential of the composite scaffold materials

Four weeks after surgery, the bone tissue surrounding the defect site was harvested, embedded in paraffin and sectioned. The expression of osteogenic markers (osteocalcin (OCN)) and a vascular marker (vascular endothelial growth factor (VEGF)) was assessed using immunofluorescence. The fluorescence intensity was quantified using ImageJ software, following the methodology described in *in vivo* analysis of the interaction between the composite materials and macrophages. The primary antibodies used were anti-OCN (GB113109; Servicebio) and anti-VEGF (GB11285; Servicebio).

### Analysis of the composite degradation mechanism *in vivo*

Transcriptomic sequencing was employed to investigate the mechanism underlying material degradation *in vivo*. Two weeks after surgery, the bone tissue surrounding the defect was excised and rinsed with precooled PBS, after which total RNA was extracted. Transcriptome sequencing (Personalbio, China) was subsequently performed to analyse the differences in the gene expression profiles between the groups. Differentially expressed genes (DEGs) were visualized in a volcano plot. KEGG pathway enrichment analysis was used to identify the signalling pathways with significant enrichment of the DEGs. The top 20 most enriched pathways were visualized using a bubble scatter plot (*P* < 0.05).

### Verification of the composite material degradation mechanism *in vivo*

Ten-week-old male Sprague–Dawley rats were randomly divided into three groups with three rats in each group. The first group was treated with the 0ZCH composite, whereas the remaining two groups were treated with the 10ZCH composite. The third group was administered the JAK/STAT pathway inhibitor ruxolitinib (GC14191; GlpBio Technology) starting on postoperative Day 1 at a dosage of 30 mg/kg twice daily for 2 weeks before sacrifice [37]. RNA and protein were extracted from the bone tissue surrounding the defect. qRT–PCR was used to measure the gene expression levels of F4/80, CD86, iNOS, Arg1 and CD206, while western blotting was used to evaluate the protein expression levels of F4/80, iNOS and Arg1.

### Statistical analysis

The data are presented as the means ± standard deviations (*n* = 3). Statistical analyses were performed using SPSS 26 (SPSS Inc., Chicago, USA) and GraphPad Prism 9 (GraphPad Software, USA). T tests were used for comparisons between two groups, whereas one-way ANOVA was used for comparisons among multiple groups and repeated measures data. A two-tailed *P* value of <0.05 was considered to indicate statistical significance. The following notations are used: *α* represents *P* < 0.05 compared with the control group, *β* represents *P* < 0.05 compared with the 0ZCH group, *γ* represents *P* < 0.05 compared with the 5ZCH group and *δ* represents *P* < 0.05 compared with the 10ZCH group.

## Results

### Characteristics of the composites

To characterize the compositions of the composites, XRD analysis was performed. The characteristic peaks of zinc silicate in the 5ZCH, 10ZCH and 15ZCH groups appeared at 2θ = 10° (Figure 1A). Additionally, FTIR spectroscopy showed that the peaks of the 0ZCH, 5ZCH, 10ZCH and 15ZCH composites were similar to those of hydroxyapatite (Figure 1B), confirming the presence of hydroxyapatite in the composites. LC-MS analysis revealed that the protein extracted from pig skin was predominantly type I collagen (Supplementary Table S1). The elemental composition of the composites was assessed through energy dispersive X-ray spectroscopy (EDS). As the proportion of zinc silicate increased, the concentrations of zinc ions and silicate structures in the composites also increased, suggesting that zinc silicate was well incorporated into the composites and that the mixing ratio was appropriate (Figure 1C). SEM revealed that the scaffold surfaces exhibited an uneven, granular texture (Figure 1D).

The average porosity of the composites, as calculated by micro-CT, is shown in Figure 1E. The mean porosity was 36.82 ± 5.29% for the 0ZCH composite, 35.32 ± 4.02% for the 5ZCH composite, 40.03 ± 3.47% for the 10ZCH composite and 38.81 ± 4.04% for the 15ZCH composite, with no significant differences among the groups (*P* > 0.05). The average pore size, displayed in Figure 1F, was 533.61 ± 57.47 μm for the 0ZCH composite, 557.29 ± 60.42 μm for the 5ZCH composite, 582.35 ± 50.95 μm for the 10ZCH composite and 562.13 ± 52.98 μm for the 15ZCH composite, with no significant differences among the groups (*P* > 0.05). These results suggest that the incorporation of zinc silicate did not affect the overall average porosity or pore size of the material, as the printing parameters (porosity and pore size) remained constant across all the groups.

As shown in Figure 1G, the compressive modulus was 0.115 ± 0.03 MPa for the 0ZCH composite, 0.232 ± 0.05 MPa for the 5ZCH composite, 0.326 ± 0.04 MPa for the 10ZCH composite and 0.464 ± 0.08 MPa for the 15ZCH composite. The compressive modulus significantly increased with increasing zinc silicate incorporation (*P* < 0.05).

### *In vitro* analysis of the interaction between the composites and macrophages

#### Macrophage polarization induced by the composites

**Figure 2:**
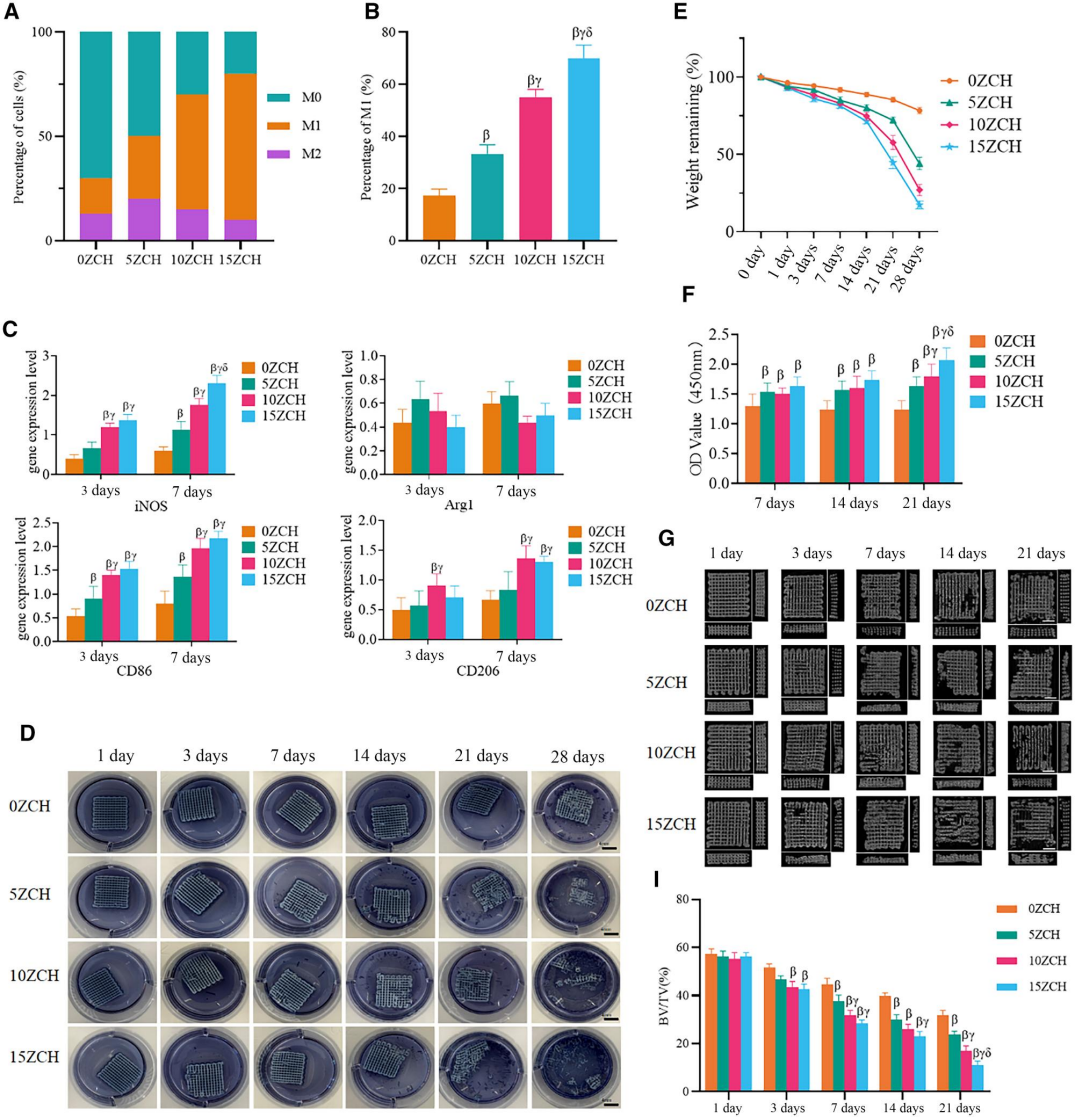
The interactions between the composites and macrophages was investigated *in vitro*. (**A**) shows the proportions of macrophages of different phenotypes in the composite groups on day 7. (**B**) shows the proportion of M1 macrophages in the different groups on day 7. (**C**) shows the gene expression levels of M1/M2 macrophage markers in each group on days 3 and 7. (**D**) shows the changes in the composites after coculture with M0 macrophages for different durations (scale bar: 4 mm). (**E**) shows the curves of the rates of residual composite material after coculture. (**F**) shows the absorbance values of the media at different times. (**G**) shows the micro-CT images captured at different times during composite degradation (scale bar: 3 mm). (**I**) shows the rates of residual composite material at different times during the degradation process. The data are presented as the mean ± SD (*n* = 3). *β* represents *P* < 0.05 compared with the 0ZCH group, *γ* represents *P* < 0.05 compared with the 5ZCH group, and *δ* represents *P* < 0.05 compared with the 10ZCH group.

To investigate the effects of the zinc silicate composites with different concentrations of zinc ions on macrophage polarization, macrophages were cultured on the composites. SEM revealed that with extended coculture duration, macrophages gradually polarized and assumed atypical morphologies corresponding to the M1 and M2 phenotypes. Live/dead staining further indicated sustained cell proliferation during coculture, with cell viability remaining at ∼95% (Supplementary Figure S1). The distribution of M0, M1 and M2 macrophages on the composites after 7 days of coculture is shown in Figure 2A, with the proportion of M0 cells decreasing from 70% to 20%. The statistical analysis of the proportion of M1 macrophages on the composites at different time points is shown in Figure 2B. As the concentration of zinc silicate increased, the proportion of M1 macrophages increased from 17% to 70%, a change that was statistically significant (*P* < 0.05).

To confirm the increase in the proportion of M1 macrophages, cells cocultured with the composites from each group were collected at different time points, and the expression levels of M1/M2-specific genes, iNOS, CD86, Arg1 and CD206, were measured (Figure 2C; Supplementary Figure S2). On Day 3, the gene expression levels of iNOS and CD86 in the zinc silicate groups were greater than those in the material group without zinc silicate. Additionally, the expression levels of all the genes were greater in the high zinc concentration treatment groups than in the 5ZCH group. The gene expression levels of iNOS and CD86 were greater on Day 7 than on Day 3, and the expression of iNOS in the 15ZCH group was significantly greater than that in the 5ZCH and 10ZCH groups (statistical details are provided in Supplementary Tables S2 and S3).

#### Effects of macrophages on composite degradation

To examine the effect of macrophages on the degradation of composites loaded with different concentrations of zinc silicate, macrophages were seeded onto the composites. An overview of composite degradation in each group at different time points is shown in Figure 2D. Over time, the elasticity of the composites gradually decreased, and the composites progressively disintegrated and lost their complete grid structure. For example, in the 15ZCH group, the residual rate decreased from 100% to 17.33 ± 2.52%. During the degradation process, the cross-linked collagen gradually dissolved in the medium, resulting in blue colouration [38]. To quantify this degradation, the residual rate of the composites after coculture was measured (Figure 2E) by collecting medium at different time points and measuring its absorbance at 450 nm (Figure 2F), which indirectly reflected the extent of composite degradation [39] (see Supplementary Table S4 and S5 for further details).

The composites were removed and freeze-dried at different time points, followed by micro-CT analysis and 3D image reconstruction to measure changes in the composite volume. The CT images of the sagittal, coronal and horizontal surfaces of the composites are shown in Figure 2G. The interior of the composites began to degrade on Day 3. Over time, the elasticity of the composites decreased, resulting in shape distortion and partial degradation, with the grid structure breaking down progressively. Quantitative analysis of the composite volume (Figure 2I) revealed that the volume of the composites in all groups gradually decreased with time. The volumes of the composites containing zinc silicate were significantly smaller than those without zinc silicate, and their degradation rate increased with increasing zinc ion concentration. Additionally, data from multiple repeated measurements at different time points were statistically analysed using one-way ANOVA, and the degradation rate in the early stage was significantly lower than that in the later stage (further details in Supplementary Table S4). The absorbance values of the culture media, as shown in Figure 2F, exhibited a similar trend (refer to Supplementary Table S5 for additional details).

### *In vivo* safety analysis

#### Haematological analysis

**Figure 3:**
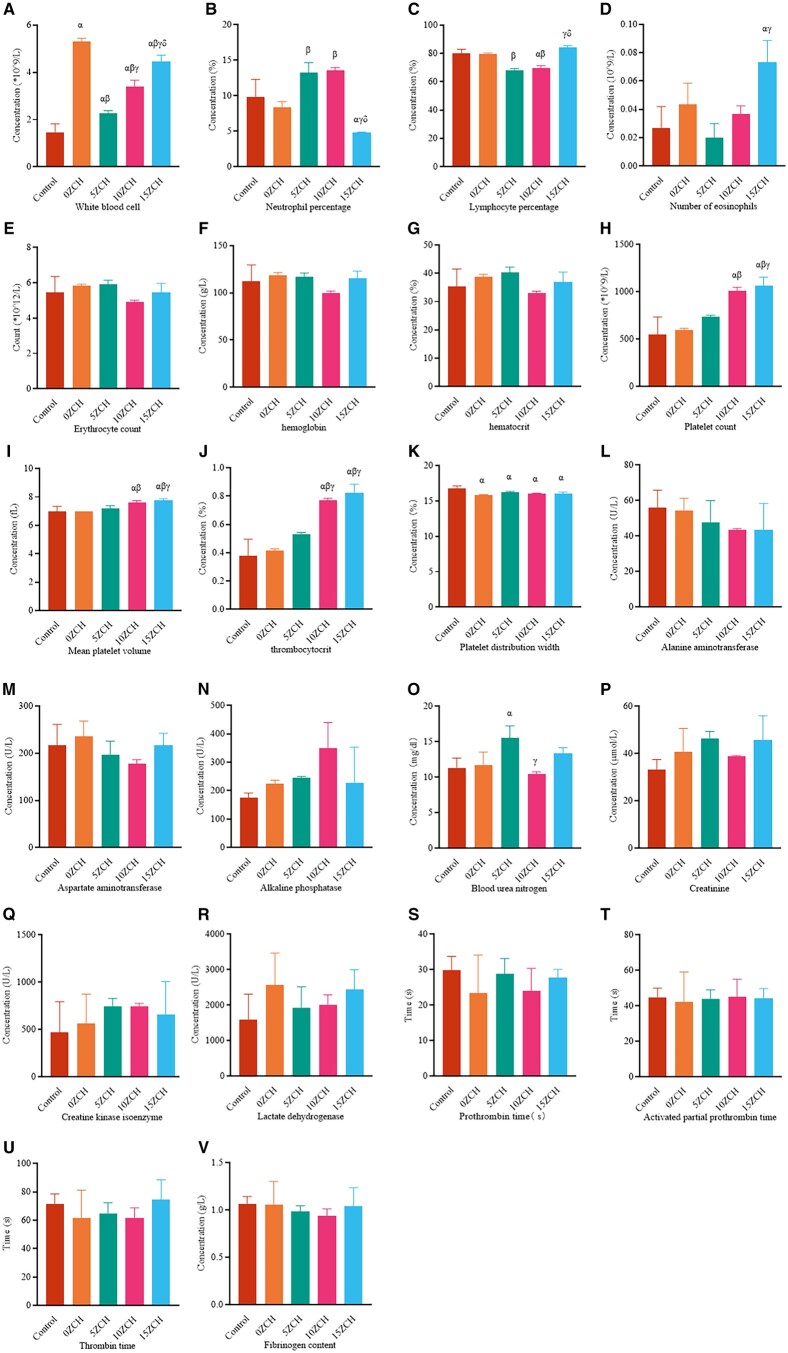
Laboratory results. (**A-V**) show the recorded white blood cell counts, neutrophil percentages, lymphocyte percentages, eosinophilic counts, erythrocyte counts, haemoglobin levels, haematocrit, platelet counts, mean platelet volume, thrombocytocrit, platelet width distribution, ALT levels, AST levels, ALP levels, UA levels, CR levels, CK-MB levels, LDH levels, prothrombin time, activated partial thromboplastin time, thrombin time, and fibrinogen levels; the data are presented as the mean ± SD (*n* = 3). *α* represents *P* < 0.05 compared with the control group, *β* represents *P* < 0.05 compared with the 0ZCH group, *γ* represents *P* < 0.05 compared with the 5ZCH group, and *δ* represents *P* < 0.05 compared with the 10ZCH group.

As foreign bodies, the implanted composites may release zinc ions into the bloodstream, potentially exerting toxic effects on the body. Therefore, routine blood parameters, liver and kidney function indicators, myocardial enzyme profiles and coagulation function were assessed in the rats 2 weeks after surgery. The results indicated that the blood parameters, liver and kidney function indicators, myocardial enzyme profiles and coagulation function parameters in all groups remained within normal ranges (Figures 3A–V), suggesting that the composites did not have any adverse effects on the rats. Further analysis revealed that the white blood cell count in the groups implanted with zinc ion-loaded composites was greater than that in the control group but lower than that in the material group without zinc silicate (Figure 3A). The proportion of neutrophils decreased with increasing zinc ion concentration in the composites (Figure 3B). There were no significant differences in red blood cell count, haemoglobin level or haematopoietic capacity among the groups (Figures 3E–G). However, the platelet count, thrombocytocrit level and average platelet volume increased with increasing zinc ion concentration in the composites (Figures 3H–J). No significant differences were observed in the other indices across the groups (further details in Supplementary Table S6).

#### Determination of the zinc ion concentration

**Figure 4:**
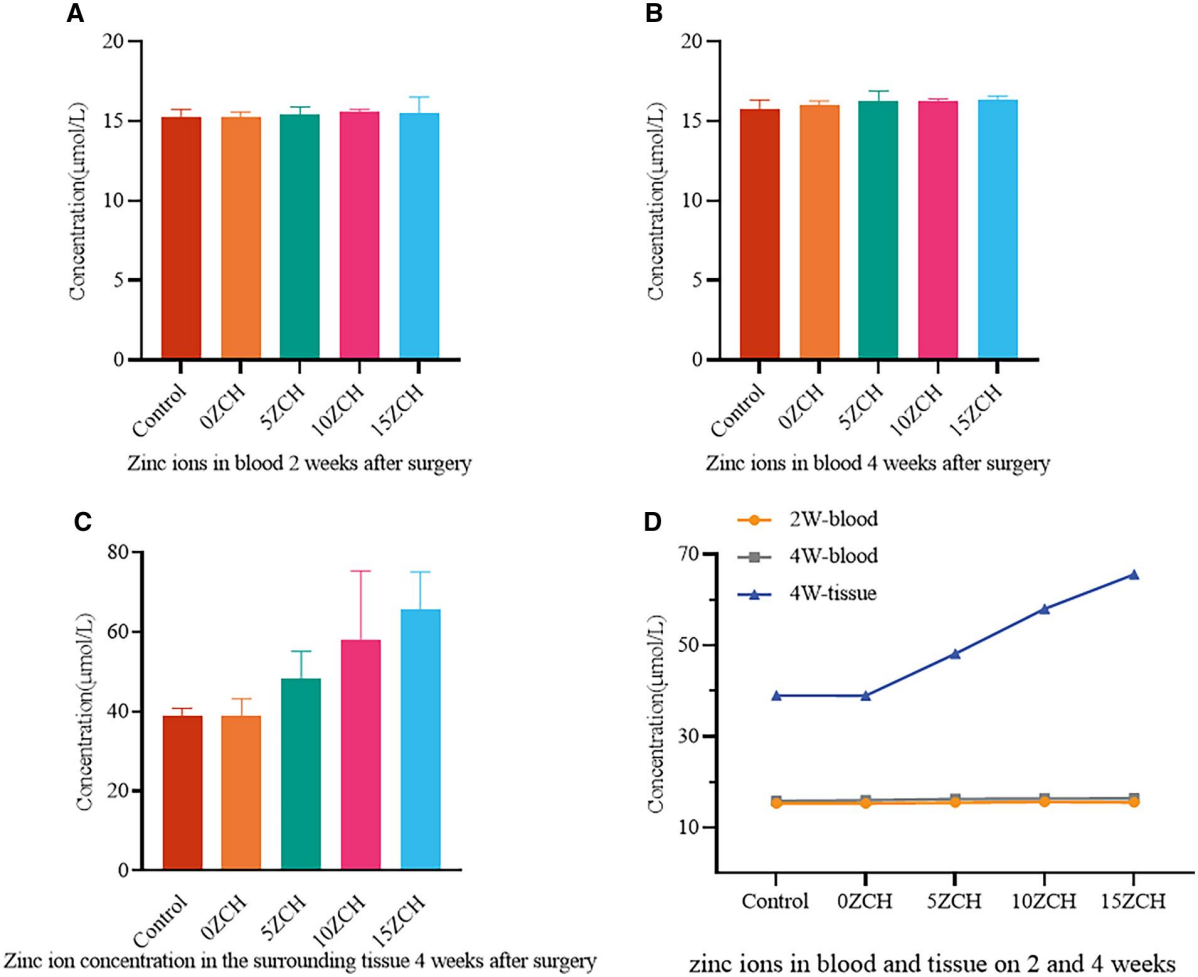
Zinc ion concentrations in blood and tissues. (**A-B**) show the concentrations of zinc ions in the blood at 2 and 4 weeks, respectively. (**C**) shows the concentration of zinc ions in the bone tissue around the bone defect at 4 weeks after surgery, as determined by inductively coupled plasma optical emission spectroscopy. (**D**) shows the curves of the zinc ion concentrations in the blood and bone tissue of the rats at different times. The data are presented as the mean ± SD (*n* = 3).

As the composites degraded, zinc ions were released into the bloodstream. Therefore, the concentration of zinc ions in the blood and bone tissue surrounding the defect was measured at different time points after surgery (Figure 4). Two and four weeks after surgery, the zinc ion concentration in the blood remained stable, with no significant difference compared with that in the control group. However, 4 weeks after surgery, the zinc ion concentration in the bone tissue around the defect was measured via ICP-OES (Figure 4C). The results revealed that the concentration of zinc ions in the bone tissue around the defect was directly proportional to the zinc concentration in the composite.

#### Histological analysis and pH and zeta potential measurements

**Figure 5:**
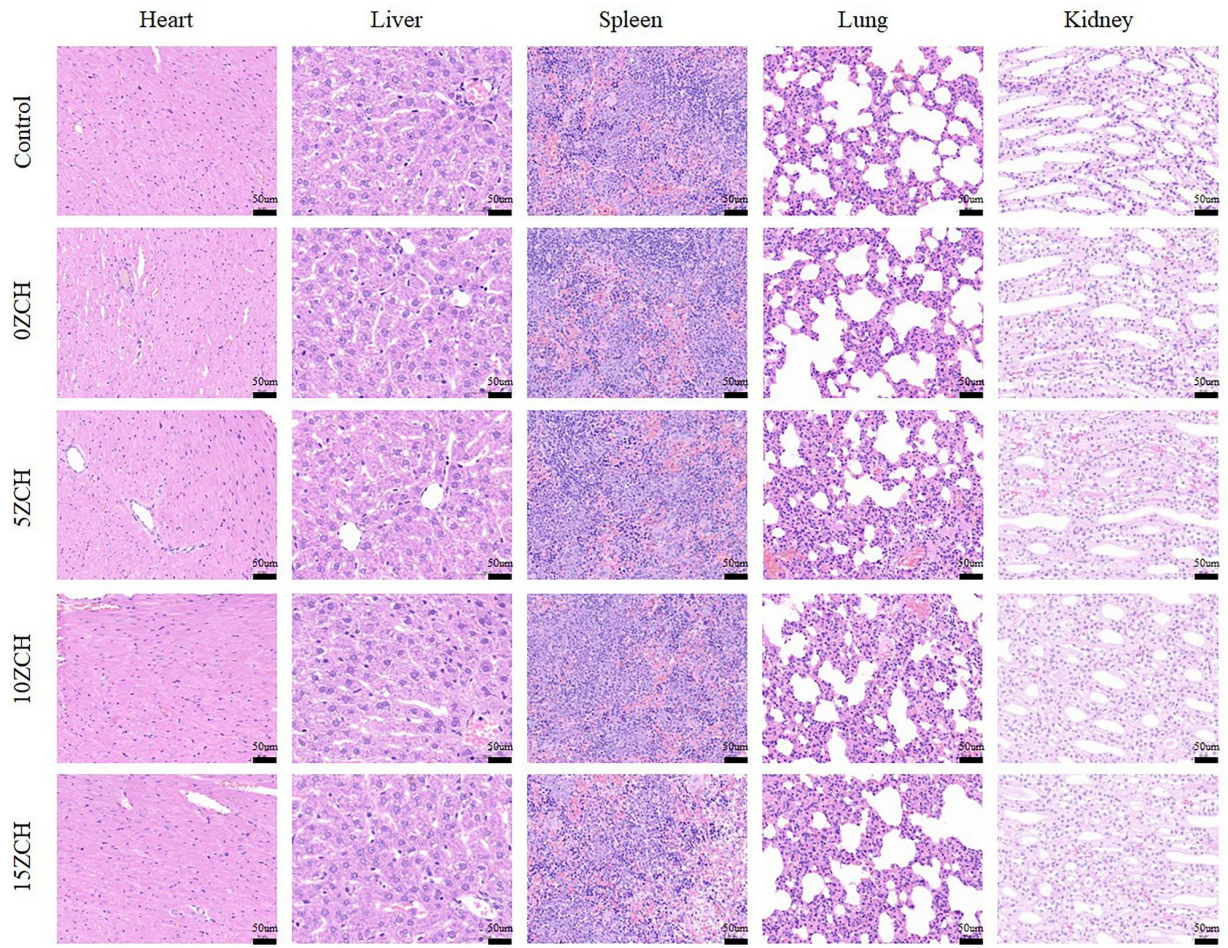
H&E staining images of heart, liver, spleen, lung and kidney tissues from rats at 4 weeks after surgery (microscope magnification: 20×; scale bar: 50 µm).

To assess the potential toxicity of the composites to organs, histopathological evaluation was performed on the heart, liver, spleen, lung, and kidney of the rats 4 weeks after surgery. H&E staining revealed no significant morphological or pathological changes, and no inflammatory cell infiltration was observed in these tissues (Figure 5). Overall, the composites demonstrated good biocompatibility and had no adverse effects on the body. The microenvironment in which the material degraded exhibited a dynamic profile of mild alkalization in terms of the pH and a slight decrease in the zeta potential 4 weeks after surgery. However, these values were not significantly different from those of the blank control group.

### Effects of the composite materials on macrophages *in vivo*

Although the composite material did not show obvious toxicity *in vitro* or *in vivo*, it remains a foreign body. Therefore, we next investigated the interaction between the composites and immune macrophages at 2 and 4 weeks after surgery. qRT–PCR analysis revealed no significant differences in the gene expression levels of F4/80 or CD68, which are specific markers of primary macrophages (M0), across all groups. Following implantation of the composites, the mRNA expression levels of iNOS and CD86, which are specific markers of M1 macrophages, increased, with the expression levels in the zinc-loaded groups being significantly greater than those in the group without zinc and the control group. The gene expression levels of iNOS and CD86 were notably greater in the 10ZCH group than in the other groups. Additionally, the expression of Arg1 and other M2 macrophage-specific markers significantly increased, with the highest expression of Arg1 observed in the 10ZCH group (Figure 6A–F; further details are provided in Supplementary Table S7). At 4 weeks after surgery, the expression levels of F4/80 and CD68 were not significantly different among the groups. However, the expression levels of iNOS, CD86, Arg1 and CD206 were greater in the zinc silicate-loaded groups than in the groups without zinc silicate. Compared with those at 2 weeks after surgery, the expression levels of iNOS and CD86 tended to decrease in the zinc silicate-loaded groups, whereas the expression levels of Arg1 and CD206 significantly increased. In contrast, these trends were not significant in the group without zinc silicate (Supplementary Figure S3).

**Figure 6:**
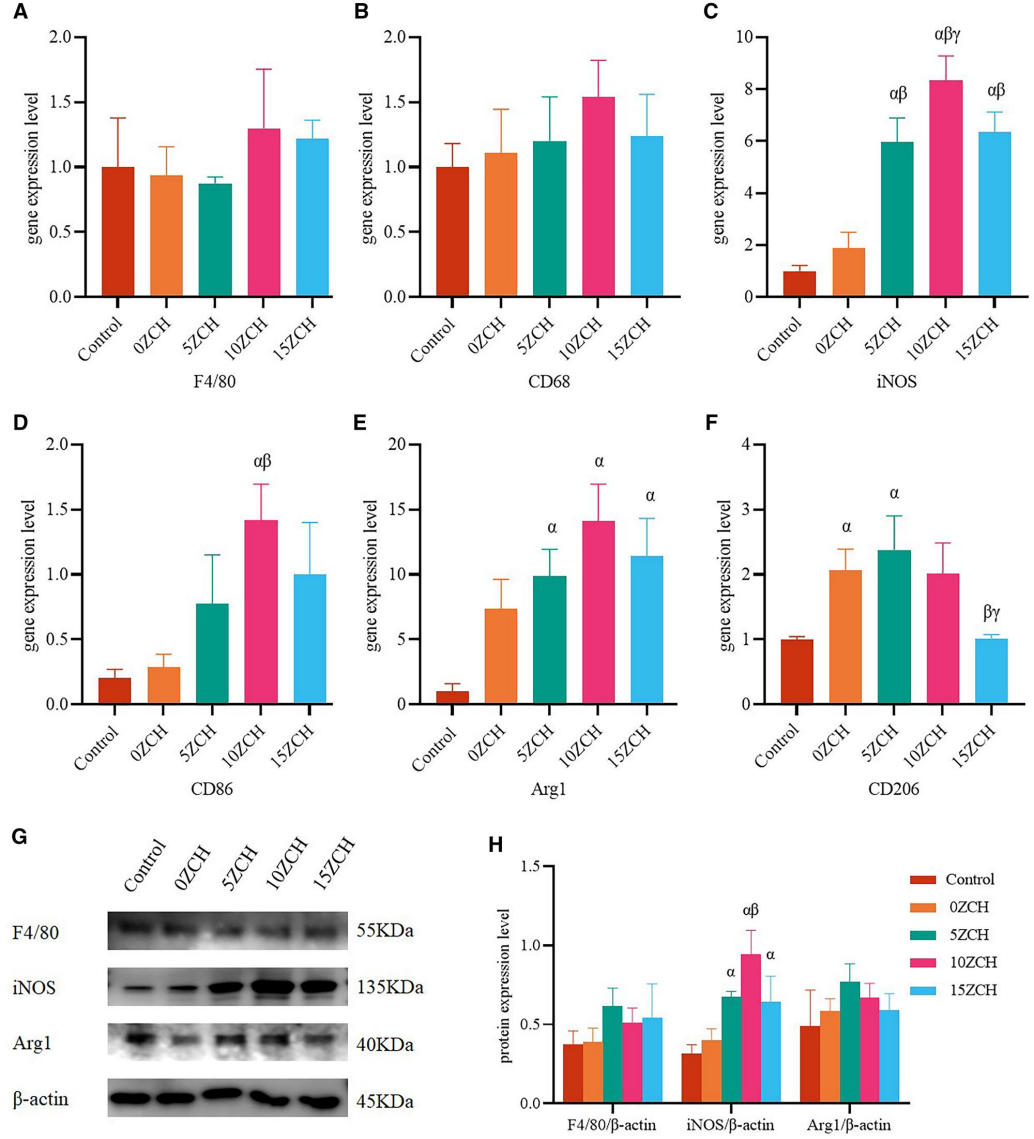
Investigation of the changes in macrophage phenotype caused by the composites *in vivo*. (**A-F**) show the gene expression levels of M0 macrophages (**A-B**), M1 macrophages (**C-D**), and M2 macrophages (**E-F**) in tissues as determined via qRT‒PCR two weeks after surgery. (**G**) shows the protein expression levels of macrophage markers in tissues measured by western blotting. (**H**) shows the quantification of protein expression by ImageJ. Immunofluorescence was used to detect the protein expression levels of markers of M0 macrophages and M1 macrophages in tissues (**I**), and quantitative analysis was conducted using ImageJ software (**K**). The protein expression levels of markers of M2 macrophages (**J**) and quantitative analysis (**L**) (magnification 20×; scale bar: 50 µm). The data are presented as the mean ± SD (*n* = 3). *α* represents *P* < 0.05 compared with the control group, *β* represents *P* < 0.05 compared with the 0ZCH group, *γ* represents *P* < 0.05 compared with the 5ZCH group, and *δ* represents *P* < 0.05 compared with the 10ZCH group.

**Figure 6:**
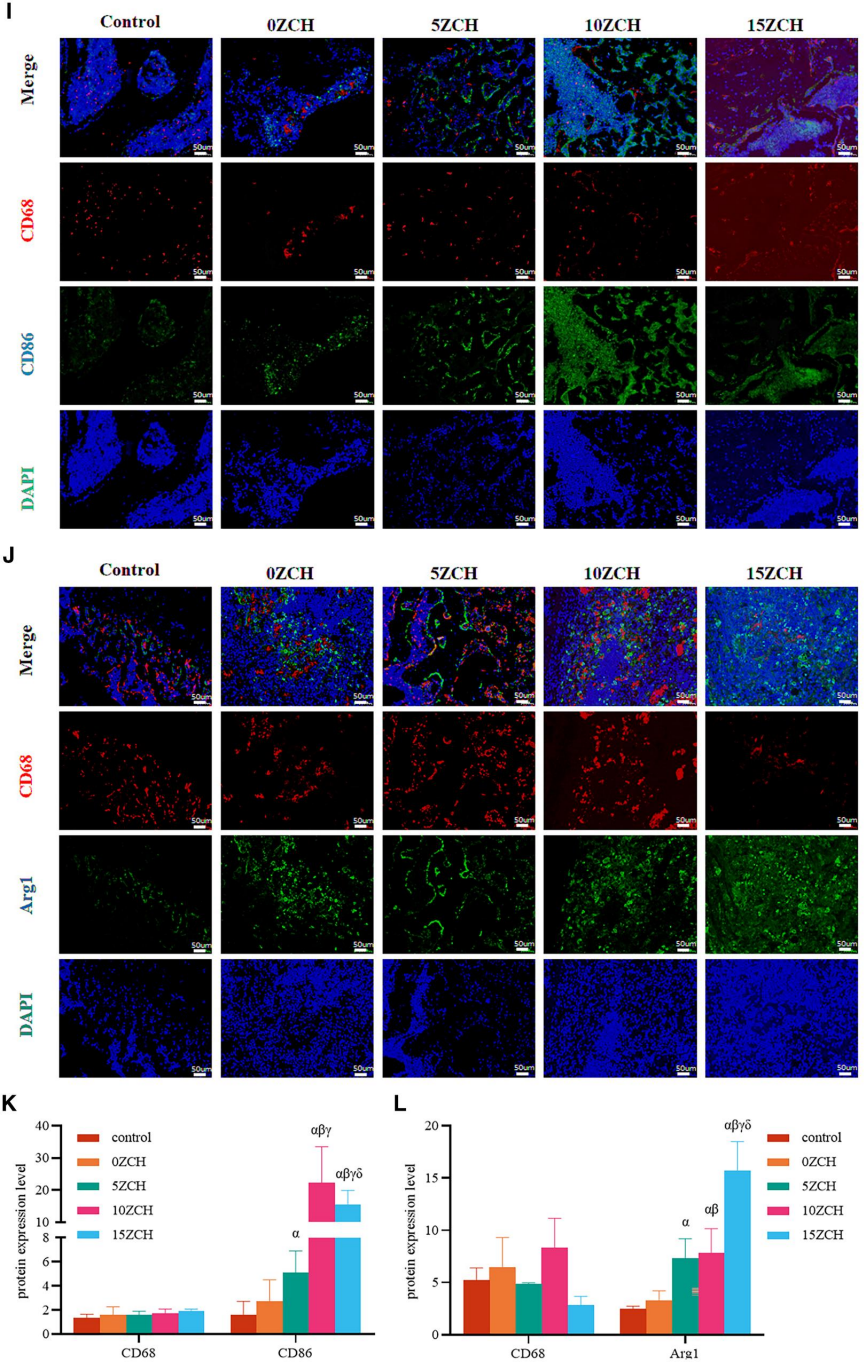
Continued.

The protein expression levels of macrophage-specific markers are shown in Figure 6G and H. The protein expression of iNOS in M1 macrophages was significantly greater than that in M0 and M2 macrophages, indicating that M1 macrophages predominated at 2 weeks after surgery. iNOS expression was significantly greater in the zinc-loaded groups than in the group without zinc, with the highest level observed in the 10ZCH group. These results suggest that zinc silicate upregulated the expression of iNOS. Compared with that in the other groups, the protein expression of F4/80 in M0 macrophages did not differ significantly across all groups, whereas the expression of Arg1 in M2 macrophages was elevated in the 5ZCH and 10ZCH groups but exhibited a decreasing tendency with increasing zinc ion concentration.

**Figure 7:**
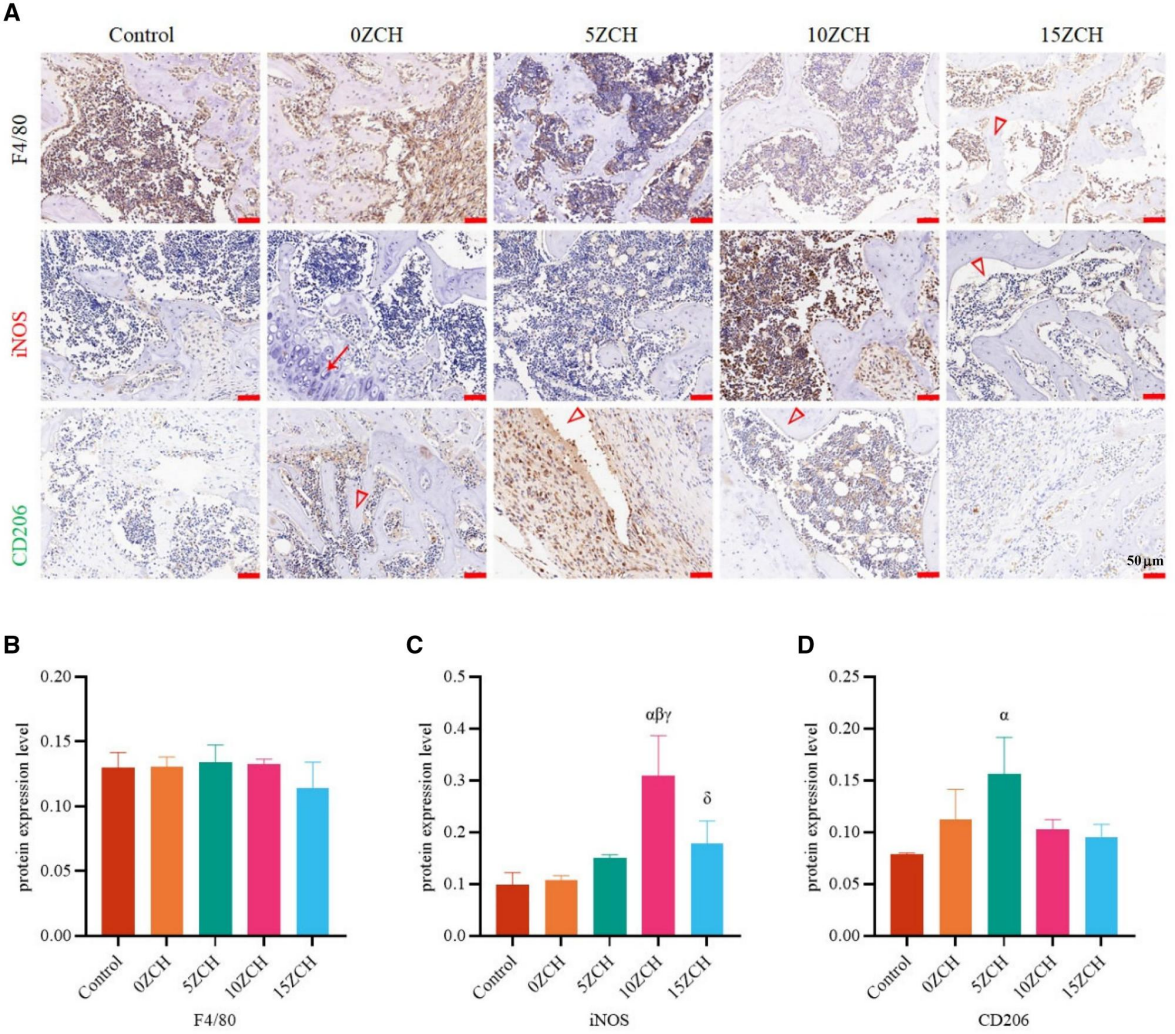
Protein expression levels of markers of different macrophage phenotypes in the bone tissue surrounding the bone defects detected by immunohistochemistry (**A**); the yellow–brown colour indicates positive staining), and quantitative analysis was performed with ImageJ software (**B-D**) (magnification, 20×; scale bar 50 µm, red arrow indicates bone tissue, Triangle indicates composites). The data are presented as the mean ± SD (*n* = 3). *α* represents *P* < 0.05 compared with the control group, *β* represents *P* < 0.05 compared with the 0ZCH group, *γ* represents P < 0.05 compared with the 5ZCH group, and *δ* represents *P* < 0.05 compared with the 10ZCH group.

Two weeks after surgery, immunofluorescence (Figure 6I–L) revealed a significant increase in the protein expression of CD86 (Figure 6I and K, green), with the highest expression observed in the 10ZCH group (CD86/CD68, 13.03). Additionally, Arg1 expression was significantly elevated in the 15ZCH group (Arg1/CD68, 5.55) (Figure 6J and L; green). The results of the immunohistochemical staining, shown in Figure 7, revealed yellow–brown spots corresponding to the target protein. There was no significant difference in F4/80 expression across all groups, but the expression of iNOS was notably greater in the zinc-loaded composite groups compared with the 0ZCH and control groups. Similarly, the protein expression of CD206 increased in the composite groups and was the highest in the 5ZCH group. However, the expression of CD206 remained lower than that of iNOS.

### Micro-CT analysis

**Figure 8:**
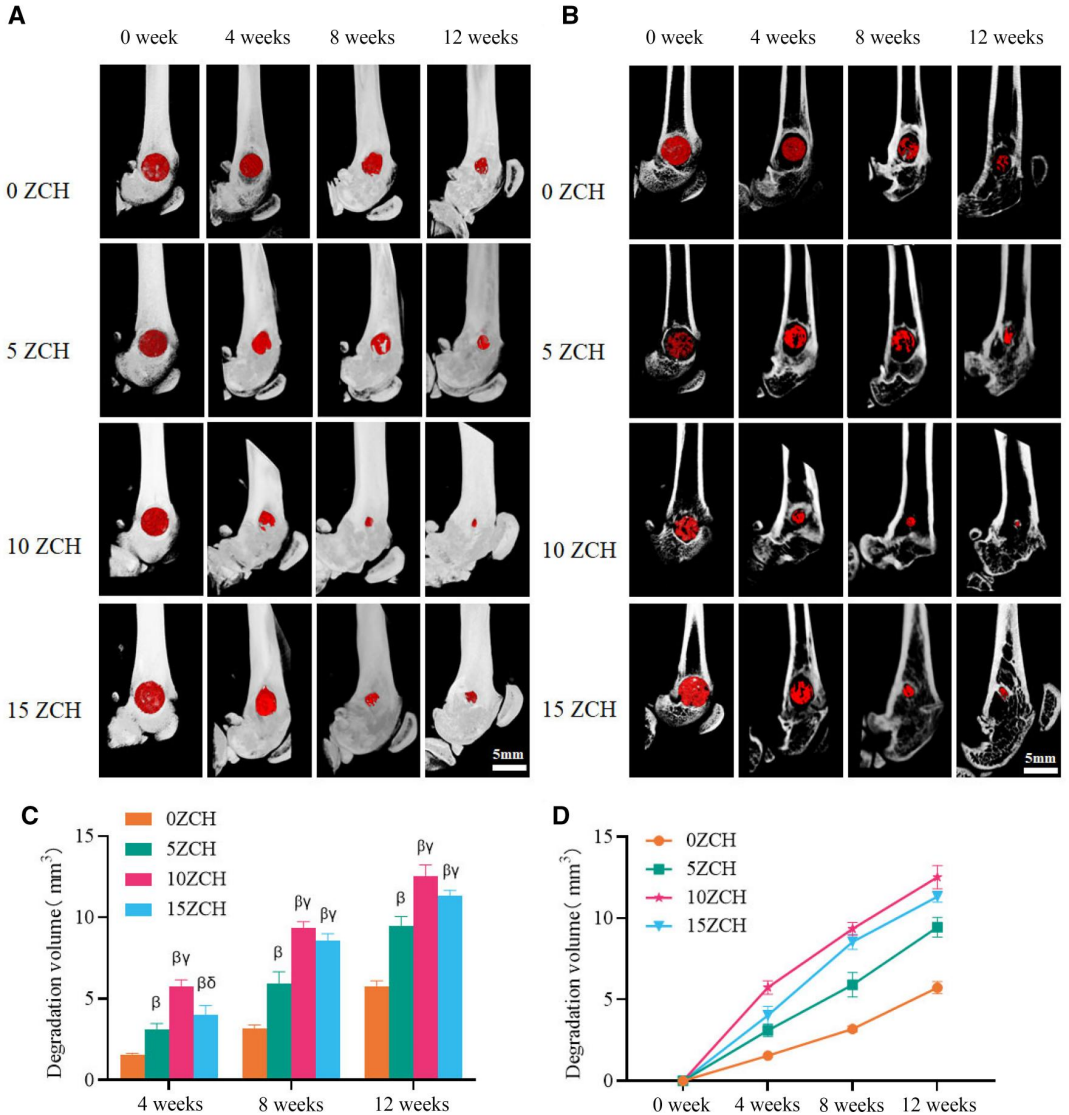
Micro-CT images showing the degradation of different composites at different times (scale bar: 5 mm). (**A**) shows the overall micro-CT images. (**B**) shows the micro-CT images of the sagittal surface. (**C**) and (**D**) show the degradation volumes of different composites *in vivo* at different times. *β* represents *P* < 0.05 compared with the 0ZCH group, *γ* represents *P* < 0.05 compared with the 5ZCH group, and *δ* represents *P* < 0.05 compared with the 10ZCH group.

The femoral condyle defect area in each rat was imaged using micro-CT at 4, 8 and 12 weeks after surgery to observe bone repair and material degradation (Figure 8). The red areas in this figure represent nondegraded material. As the implantation duration increased, the materials exhibited varying degrees of degradation. In both the surface plane and the sagittal plane, the 10ZCH group had the least remaining implant material (Figure 8A and B). Quantitative analysis confirmed that the greatest material degradation occurred in the 10ZCH group, followed by the 15ZCH group, while the degradation of the 5ZCH composite was significantly greater than that of the material without zinc. Further analysis of the 10ZCH group revealed a steeper degradation slope during the early stages of bone repair than during the later stages, with the difference being statistically significant (Figure 8C and D; see Supplementary Table S8 for additional details).

### Investigation of the early osteogenic activity of the composite scaffolds *in vivo*

To assess the osteogenic and angiogenic potential of the composite scaffold materials, we performed H&E staining and immunofluorescence staining of the bone tissue surrounding the defect 4 weeks after surgery.

**Figure 9:**
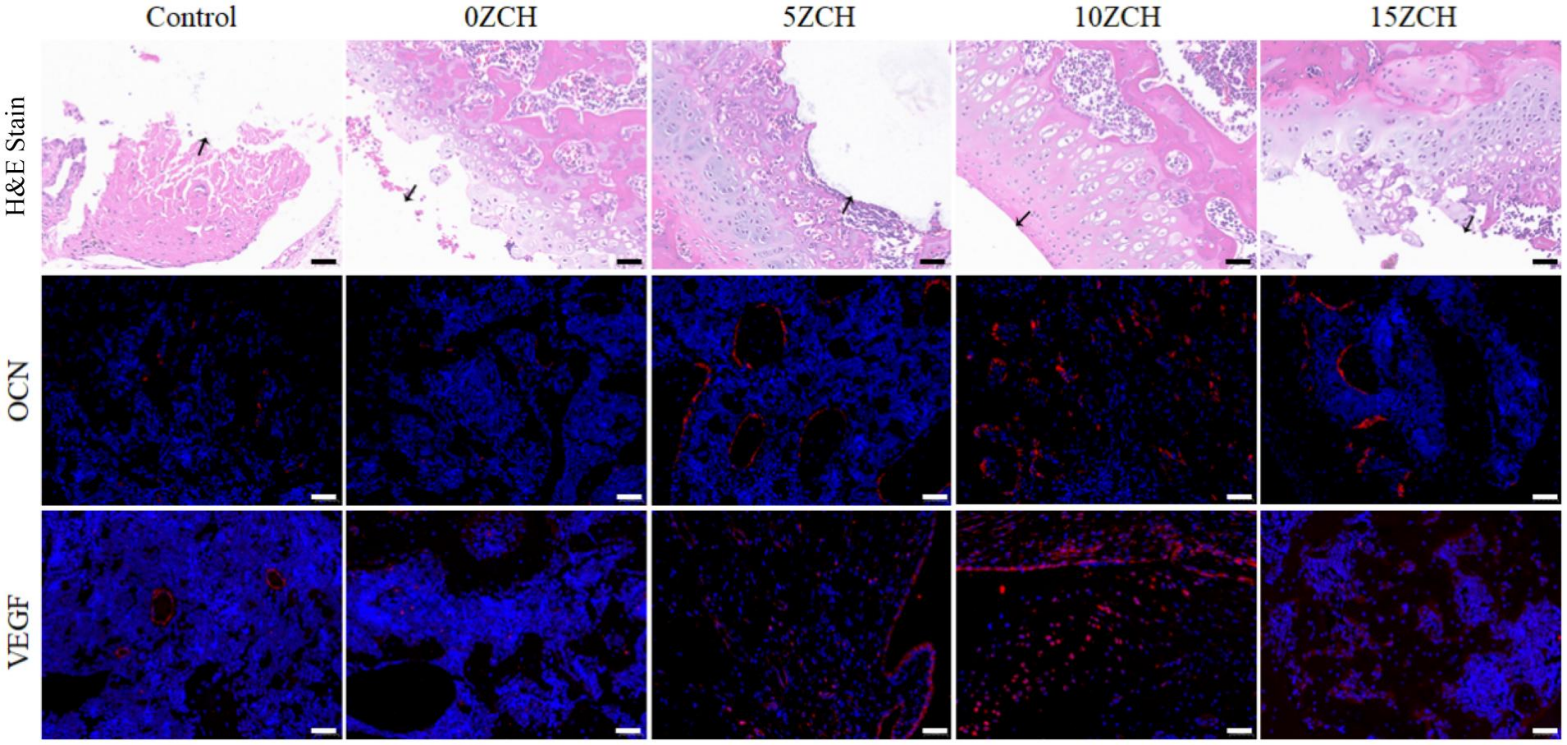
H&E staining of bone tissue around the defect and immunofluorescence of OCN and VEGF (black arrows indicate the boundary between the material implant and the surrounding newly formed bone tissue. Magnification 20×, scale bar: 50 μm). H&E staining revealed that the 10ZCH group exhibited significantly higher osteogenesis and vascular density compared to the other groups. Similarly, immunofluorescence analysis demonstrated that the expression levels of OCN and VEGF were the highest in the 10ZCH group.

H&E staining revealed that the densities of newly formed trabecular bone and microvessels in the defect area did not differ significantly between the blank control group and the non-zinc silicate group. However, these parameters significantly increased in the zinc silicate-loaded groups (Figure 9).

Immunofluorescence staining revealed a similar trend. As depicted in Figure 9, the expression levels of the osteogenic marker OCN and the angiogenic marker VEGF were extremely low in the blank control and non-zinc silicate groups. In contrast, their expression was significantly upregulated in the zinc silicate-loaded groups.

### Elucidation and validation of the mechanism through which the composites are degraded *in vivo*

**Figure 10:**
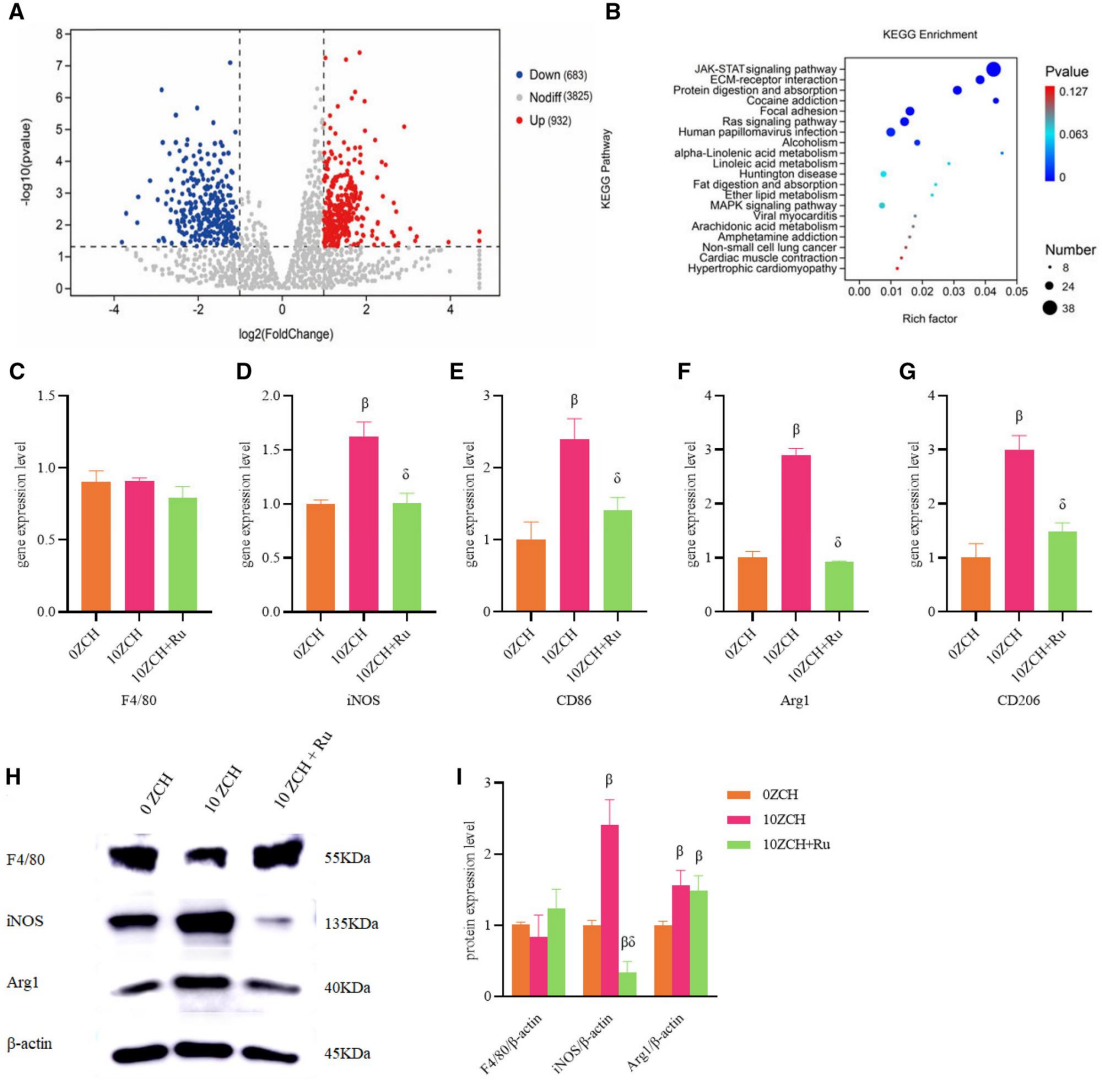
Mechanism of composite degradation *in vivo*. (**A**) and (**B**) show the transcriptome sequencing results of the bone tissue surrounding the defect (volcano plot of the differentially expressed genes and KEGG enrichment analysis). (**C-G**) show the gene expression levels of markers of macrophages of different phenotypes detected by qRT‒PCR after the administration of ruxolitinib. Moreover, western blotting was performed to detect the expression levels of specific marker proteins of macrophages of different phenotypes in bone tissues around the bone defect (**H**), and quantitative analysis was conducted with ImageJ software (**I**). The data are presented as the mean ± SD (*n* = 3). *β* represents *P* < 0.05 compared with the 0ZCH group, and *δ* represents *P* < 0.05 compared with the 10ZCH group.

Previous experiments revealed that the 10ZCH composite exhibited the most significant degradation *in vivo*. To investigate the underlying mechanism of composite degradation, transcriptome sequencing was conducted on samples from both the 10ZCH and 0ZCH groups. The gene expression profile of the zinc-loaded composite group significantly differed from that of the non-zinc composite group (Figure 10A). To further explore the signalling pathways involved in composite degradation, Kyoto Encyclopedia of Genes and Genomes (KEGG) pathway analysis was performed. The results revealed that the JAK/STAT pathway, which is involved in the regulation of macrophage polarization, was significantly enriched in the upregulated genes (Figure 10B). Combining these findings with previous data, we observed that the proportion of M1 macrophages in the zinc-loaded composite group increased during the early postoperative stage and that the degradation rate was faster than that in the group without zinc. To verify the role of the JAK/STAT pathway in macrophage polarization, we treated the samples with ruxolitinib, a JAK/STAT pathway inhibitor and performed qRT–PCR and western blotting. The results revealed significant decreases in the gene and protein expression levels of iNOS after ruxolitinib treatment (Figure 10C–I) (see Supplementary Table S9 for details).

## Discussion

Bone is primarily composed of cells, fibres and stroma [1]. The healing of bone defects involves complex physiological processes that are coordinated across multiple systems. These processes include the formation of a haematoma, the development of a primitive callus and the subsequent transformation and shaping of the callus. Rather than being isolated, these stages are interconnected and interdependent [40]. With growing interest in osteoimmunomodulation, biodegradable bone substitutes have garnered increasing attention from researchers.

In recent years, extensive and in-depth research has been conducted on degradable bone repair materials for diabetic patients. It is widely recognized that an ideal material should not only possess antioxidant and anti-infective properties but also synergistically modulate immune responses and promote vascular and neural regeneration to ultimately achieve efficient bone regeneration [30, 41]. However, significant gaps remain in recent studies: on the one hand, the spatiotemporal matching of the material degradation rate and the rate of new bone formation is still unclear; on the other hand, systematic exploration of the degradation behaviour of materials in complex microenvironments, such as in diabetes and trauma patients, and the underlying regulatory mechanisms, is lacking.

In this study, ZCH composites were synthesized. XRD revealed that the characteristic peaks of the composites with varying concentrations of zinc silicate were similar to those of pure zinc silicate. FTIR spectroscopy demonstrated that all composites displayed the characteristic peaks of hydroxyapatite, whereas energy spectrum analysis confirmed that the zinc silicate concentration in the composites was directly proportional to the amount of zinc silicate added. This suggests that the materials contained hydroxyapatite along with varying amounts of zinc silicate and that the materials were uniformly mixed, indicating successful preparation of the composites. Additionally, although the zinc silicate concentration did not significantly affect the porosity or pore size of the scaffolds, the compressive modulus increased proportionally with zinc silicate content. The material degradation microenvironment exhibited a dynamic profile of mild alkalization with respect to the pH and a slight decrease in the zeta potential. However, these values were not significantly different from those in the control group.

The immune response after biomaterial implantation plays a crucial role in determining its fate. Most previous studies have focused on developing biomaterials that promote the osteogenic differentiation of stem cells, as well as vascularization and neurogenesis [30, 41]. However, before osteoblasts can reach the defect and begin forming bone, the inflammatory response must be resolved. Persistent activation of the immune system can lead to chronic inflammation, potentially resulting in the formation of a fibrous capsule around the implant. On the other hand, the absence of an inflammatory response can hinder material integration, resulting in residual tissue debris near the defect. The initial response of the host to biomaterials is primarily controlled by macrophages and the factors they secrete. Macrophages play pivotal roles in multiple stages of bone repair and exhibit distinct phenotypes, including the proinflammatory M1 phenotype and the anti-inflammatory M2 phenotype. These two macrophage states represent extremes, but they can coexist and interconvert, which is essential for both bone healing and biomaterial integration [42].

At the beginning of the repair process, macrophages are predominantly in the M0 state. Over time, however, the number of M1 macrophages increases. Interestingly, in the groups treated with composites loaded with zinc silicate, the degradation of the composites was more pronounced than in groups treated with composites without zinc silicate. Moreover, as the concentration of zinc silicate in the composite increased, the proportion of M1 macrophages also increased, leading to greater mass loss and a reduction in the residual amount of implanted material. We previously showed that zinc silicate improves the mechanical properties of composites, increases their surface roughness and promotes cell adhesion. This phenomenon may be attributed to the slow release of zinc ions into the surrounding media, which influences the polarization of macrophages as the composites degrade. These M1 macrophages, in turn, further accelerate the degradation of the composite material. To support this hypothesis, qRT–PCR was performed on cocultured cells, which revealed that the expression levels of iNOS and CD86 increased over time with increasing concentrations of zinc silicate. These results suggest that zinc silicate promotes M1 macrophage polarization in a concentration-dependent manner.

Liu et al. reported that with increasing zinc ion concentration, the expression of iNOS and CD86 was downregulated in the Zn4 and Zn20 groups but upregulated in the Zn100 group. In contrast, the expression levels of Arg1 and CD206 consistently decreased across these groups [43]. Another study revealed that incorporating 30 mol% zinc into calcium silicate promoted the recruitment of macrophages and facilitated M2 macrophage polarization while simultaneously inhibiting M1 macrophage polarization [44]. These conflicting results suggest that the phenotypic changes in macrophages may be influenced by the concentration of zinc silicate, highlighting the concentration-dependent effects of zinc on macrophage polarization.

Two weeks after the operation, routine blood and biochemical tests were conducted on the peripheral blood of the rats (Figure 3), and all the indicators were within normal ranges. However, compared with that in the control group, the number of white blood cells in the 15ZCH group increased, whereas the proportion of neutrophils decreased significantly. This finding aligns with previous reports that suggest that zinc-containing composites may regulate immune responses and inhibit inflammation [45, 46]. Moreover, the number, volume and accumulation of platelets increased with increasing zinc ion concentration in the composites. This likely reflects the activation of the JAK/STAT pathway and an increase in thrombopoietin (TPO) levels [47, 48]. There were no significant abnormalities in liver function, renal function, coagulation function or the myocardial enzyme profile. Histological examination via H&E staining of heart, liver, spleen, lung and kidney tissues from rats in the composite-treated groups (Figure 4) revealed no signs of mitotic proliferation, nuclear pyknosis, nucleolysis or inflammatory cell infiltration compared with the major organs in the control group.

Although the concentration of zinc ions in the tissue surrounding the bone defect increased with increasing zinc ion concentration in the composites at 4 weeks after surgery, it remained below the threshold (79.5 µmol/L) that could inhibit osteoblast formation [49]. Furthermore, the zinc ion concentration in the blood remained within the normal range at both 2 and 4 weeks after surgery, with no significant difference among the groups. This observation is consistent with prior findings that zinc ions are primarily stored in skeletal muscle and bone, with only a small fraction circulating in the blood [17, 50]. Additionally, numerous studies have indicated that zinc promotes osteoblast differentiation and inhibits osteoclast differentiation, whereas zinc deficiency can lead to bone loss, growth retardation and other bone abnormalities [19]. Collectively, these results demonstrate that the composites loaded with <0.337 M zinc silicate exhibit favourable safety and histocompatibility *in vivo*.

To investigate the early osteoimmunomodulatory functions of these composites *in vivo*, we first examined their effects on macrophages. qRT–PCR revealed that the expression levels of M1 macrophage markers were significantly greater in the zinc-loaded groups than in both the 0ZCH group and the control group at 2 weeks after surgery. Notably, no significant difference was observed between the 0ZCH group and the control group, indicating that the zinc silicate released from the composite material primarily influenced macrophage polarization, which aligns with findings from previous studies [51, 52]. Further analysis confirmed that the proportion of M1 macrophages was greatest in the 10ZCH group. These results were consistent across the western blotting, immunofluorescence and immunohistochemistry experiments. At 4 weeks after surgery, the proportion of M1 macrophages tended to decrease in the groups treated with zinc silicate-loaded composites, whereas the proportion of M2 macrophages increased significantly. In contrast, the non-zinc silicate-loaded group showed no significant differences in the percentages of both M1 and M2 macrophages compared with that observed 2 weeks after surgery. These results indicate that zinc silicate promotes the polarization of macrophages towards the proinflammatory M1 phenotype in the early postoperative phase. Over time, zinc silicate further facilitates an earlier transition of macrophages from the M1 phenotype to the anti-inflammatory M2 phenotype, thereby shortening the duration of inflammation and accelerating the initiation of osteogenesis. However, these findings contrast with those of the *in vitro* studies, likely because of the more complex microenvironment *in vivo*. Overall, our results suggest that the release of zinc ions from the composites promotes M1 macrophage polarization during the early postoperative period, accelerates macrophage polarization from the M1 phenotype to the M2 phenotype, decreases the duration of inflammation and facilitates earlier initiation of tissue repair.

The micro-CT results revealed that the degradation of the zinc-loaded materials was greater than that of the 0ZCH composite at various postoperative stages. Among the zinc-loaded groups, the greatest degradation was noted in the 10ZCH group, with the least amount of residual implant debris. This finding aligns with the greater expression of M1 macrophage markers observed in the zinc-loaded groups during the early postoperative period. Previous studies have shown that zinc ions released from composites promote the polarization of M1 macrophages, which, in turn, secrete matrix metalloproteinases (MMPs) such as MMP2 and MMP9. These MMPs facilitate the degradation of collagen and other extracellular matrix (ECM) components, thereby accelerating composite degradation [12]. The degradation rate in the 10ZCH group was significantly greater during the first month than during the following 2 months, likely because of the predominance of proinflammatory M1 macrophages in the early phase. Over time, the number of M1 macrophages decreased, and the M1 macrophages gradually polarized into anti-inflammatory M2 macrophages. Furthermore, our investigation of the osteogenic and angiogenic properties of the zinc silicate composite materials revealed that the 10ZCH group demonstrated superior bone formation and vascularization potential. This finding supports our earlier work, in which we identified the P38/MAPK pathway as a key regulator of monocyte function [27].

Transcriptome sequencing revealed significant enrichment of the JAK/STAT pathway in the 10ZCH group compared with the 0ZCH group. The inhibition of this pathway with a specific inhibitor led to the significant downregulation of both iNOS (an M1 macrophage marker) and Arg1 (an M2 macrophage marker), with a more pronounced decrease in iNOS expression. This finding suggests that in our model, the JAK/STAT pathway is critically involved in early proinflammatory (M1) macrophage polarization, which is consistent with its role in initiating immune responses [53–55].

The limitations of this study are as follows. (i) The temporal regulation of M1 macrophage polarization by zinc ions has not been fully explored. An excessively prolonged or intense proinflammatory response could lead to the formation of a biofilm around the material, potentially hindering bone repair. (ii) While we previously investigated the osteogenic properties of this material during osseointegration, this study focused primarily on the proinflammatory M1 macrophage response during composite degradation. The role of anti-inflammatory M2 macrophages was not examined in depth. (iii) Sprague–Dawley rats were used in this study. To better facilitate clinical translation, further evaluation over longer durations in large animal models is needed. These limitations highlight areas for future research.

## Conclusion

In the early postoperative stage, the zinc silicate composite promotes its own degradation and orchestrates macrophage polarization via the JAK/STAT pathway. This activity shortens the inflammatory phase and expedites the subsequent initiation of tissue repair. The composite demonstrates favourable histocompatibility and controllable degradability *in vivo*. To advance its clinical translation, future studies will involve large animal experiments, positioning this material as a promising new strategy for treating segmental bone defects.

## Supplementary Material

rbag037_Supplementary_Data

## Data Availability

The data are available from the authors upon reasonable request.

## References

[1] Wei S , Ma JX, Xu L, Gu XS, Ma XL. Biodegradable materials for bone defect repair. Mil Med Res 2020;7:54. 33172503 10.1186/s40779-020-00280-6 PMC7653714

[2] Zhang L , Yang G, Johnson BN, Jia X. Three-dimensional (3D) printed scaffold and material selection for bone repair. Acta Biomater 2019;84:16–33. 30481607 10.1016/j.actbio.2018.11.039

[3] Habibovic P. Strategic directions in osteoinduction and biomimetics. Tissue Eng Part A 2017;23:1295–6. 29032745 10.1089/ten.TEA.2017.0430

[4] Baldwin P , Li DJ, Auston DA, Mir HS, Yoon RS, Koval KJ. Autograft, allograft, and bone graft substitutes: clinical evidence and indications for use in the setting of orthopaedic trauma surgery. J Orthop Trauma 2019;33:203–13. 30633080 10.1097/BOT.0000000000001420

[5] Ward BB , Brown SE, Krebsbach PH. Bioengineering strategies for regeneration of craniofacial bone: a review of emerging technologies. Oral Dis 2010;16:709–16. 20534013 10.1111/j.1601-0825.2010.01682.x

[6] Sheikh Z , Najeeb S, Khurshid Z, Verma V, Rashid H, Glogauer M. Biodegradable materials for bone repair and tissue engineering applications. Materials (Basel) 2015;8:5744–94. 28793533 10.3390/ma8095273 PMC5512653

[7] Guo H , Xia D, Zheng Y, Zhu Y, Liu Y, Zhou Y. A pure zinc membrane with degradability and osteogenesis promotion for guided bone regeneration: in vitro and in vivo studies. Acta Biomater 2020;106:396–409. 32092431 10.1016/j.actbio.2020.02.024

[8] Arron JR , Choi Y. Bone versus immune system. Nature 2000;408:535–6. 10.1038/35046196 11117729

[9] Takayanagi H. Osteoimmunology: shared mechanisms and crosstalk between the immune and bone systems. Nat Rev Immunol 2007;7:292–304. 17380158 10.1038/nri2062

[10] Jain N , Moeller J, Vogel V. Mechanobiology of macrophages: how physical factors coregulate macrophage plasticity and phagocytosis. Annu Rev Biomed Eng 2019;21:267–97. 31167103 10.1146/annurev-bioeng-062117-121224

[11] Murray PJ. On macrophage diversity and inflammatory metabolic timers. Nat Rev Immunol 2020;20:89–90. 31804612 10.1038/s41577-019-0260-2

[12] Murray PJ , Wynn TA. Protective and pathogenic functions of macrophage subsets. Nat Rev Immunol 2011;11:723–37. 21997792 10.1038/nri3073 PMC3422549

[13] Boraschi D , Italiani P. Immunosenescence and vaccine failure in the elderly: strategies for improving response. Immunol Lett 2014;162:346–53. 24960535 10.1016/j.imlet.2014.06.006

[14] Italiani P , Boraschi D. From monocytes to M1/M2 macrophages: phenotypical vs. functional differentiation. Front Immunol 2014;5:514. 25368618 10.3389/fimmu.2014.00514 PMC4201108

[15] Piccolo V , Curina A, Genua M, Ghisletti S, Simonatto M, Sabo A, Amati B, Ostuni R, Natoli G. Opposing macrophage polarization programs show extensive epigenomic and transcriptional cross-talk. Nat Immunol 2017;18:530–40. 28288101 10.1038/ni.3710 PMC5524187

[16] Wang Y , Wang X, Pang Y, Li X, Gao C, Zhang D, Li G, Yu Y, Yang X, Cai Q. Ion‐engineered microcryogels via osteogenesis‐angiogenesis coupling and inflammation reversing augment vascularized bone regeneration. Adv Funct Materials 2024;34:1–15.

[17] Chasapis CT , Ntoupa PA, Spiliopoulou CA, Stefanidou ME. Recent aspects of the effects of zinc on human health. Arch Toxicol 2020;94:1443–60. 32394086 10.1007/s00204-020-02702-9

[18] Chen K , Zhou G, Li Q, Tang H, Wang S, Li P, Gu X, Fan Y. In vitro degradation, biocompatibility and antibacterial properties of pure zinc: assessing the potential of Zn as a guided bone regeneration membrane. J Mater Chem B 2021;9:5114–27. 34128016 10.1039/d1tb00596k

[19] Dermience M , Lognay G, Mathieu F, Goyens P. Effects of thirty elements on bone metabolism. J Trace Elem Med Biol 2015;32:86–106. 26302917 10.1016/j.jtemb.2015.06.005

[20] Qiao Y , Zhang W, Tian P, Meng F, Zhu H, Jiang X, Liu X, Chu PK. Stimulation of bone growth following zinc incorporation into biomaterials. Biomaterials 2014;35:6882–97. 24862443 10.1016/j.biomaterials.2014.04.101

[21] Yamaguchi M. Role of zinc in bone formation and bone resorption. J Trace Elem Exp Med 1998;11:119–35.

[22] Fraker PJ , King LE. Reprogramming of the immune system during zinc deficiency. Annu Rev Nutr 2004;24:277–98. 15189122 10.1146/annurev.nutr.24.012003.132454

[23] Wessels I , Maywald M, Rink L. Zinc as a gatekeeper of immune function. Nutrients 2017;9:1–44. 10.3390/nu9121286 PMC5748737 29186856

[24] Shao X , Wang X, Xu F, Dai T, Zhou JG, Liu J, Song K, Tian L, Liu B, Liu Y. In vivo biocompatibility and degradability of a Zn-Mg-Fe alloy osteosynthesis system. Bioact Mater 2022;7:154–66. 34466724 10.1016/j.bioactmat.2021.05.012 PMC8379423

[25] Bai X , Liu W, Xu L, Ye Q, Zhou H, Berg C, Yuan H, Li J, Xia W. Sequential macrophage transition facilitates endogenous bone regeneration induced by Zn-doped porous microcrystalline bioactive glass. J Mater Chem B 2021;9:2885–98. 33721004 10.1039/d0tb02884c

[26] Gao H , Dai W, Zhao L, Min J, Wang F. The role of zinc and zinc homeostasis in macrophage function. J Immunol Res 2018;2018:6872621. 30622979 10.1155/2018/6872621 PMC6304900

[27] Song Y , Wu H, Gao Y, Li J, Lin K, Liu B, Lei X, Cheng P, Zhang S, Wang Y, Sun J, Bi L, Pei G. Zinc silicate/nano-hydroxyapatite/collagen scaffolds promote angiogenesis and bone regeneration via the p38 MAPK pathway in activated monocytes. ACS Appl Mater Interfaces 2020;12:16058–75. 32182418 10.1021/acsami.0c00470

[28] El-Rashidy AA , Roether JA, Harhaus L, Kneser U, Boccaccini AR. Regenerating bone with bioactive glass scaffolds: a review of in vivo studies in bone defect models. Acta Biomater 2017;62:1–28. 28844964 10.1016/j.actbio.2017.08.030

[29] Tan S , Zeng H, Li W, Liu H, Gu X, Luo X, Zhao X. Copper nanocluster‐decorated magnesium silicate‐based microneedle enhances antimicrobial effects and tissue remodeling for diabetic wounds. Small Sci 2026;6:e202500442. 41537187 10.1002/smsc.202500442 PMC12798781

[30] Wu M , Liu H, Zhu Y, Wu P, Chen Y, Deng Z, Zhu X, Cai L. Bioinspired soft-hard combined system with mild photothermal therapeutic activity promotes diabetic bone defect healing via synergetic effects of immune activation and angiogenesis. Theranostics 2024;14:4014–57. 38994032 10.7150/thno.97335 PMC11234279

[31] Wu M , Liu H, Li D, Zhu Y, Wu P, Chen Z, Chen F, Chen Y, Deng Z, Cai L. Smart‐responsive multifunctional therapeutic system for improved regenerative microenvironment and accelerated bone regeneration via mild photothermal therapy. Advanced Science 2024;11:1–28. 10.1002/advs.202304641 PMC10787108 37933988

[32] Zhu Y , Liu H, Wu P, Chen Y, Deng Z, Cai L, Wu M. Multifunctional injectable hydrogel system as a mild photothermal-assisted therapeutic platform for programmed regulation of inflammation and osteo-microenvironment for enhanced healing of diabetic bone defects in situ. Theranostics 2024;14:7140–98. 39629118 10.7150/thno.102779 PMC11610133

[33] Xiong K , Wu T, Fan Q, Chen L, Yan M. Novel reduced graphene oxide/zinc silicate/calcium silicate electroconductive biocomposite for stimulating osteoporotic bone regeneration. ACS Appl Mater Interfaces 2017;9:44356–68. 29211449 10.1021/acsami.7b16206

[34] Taga Y , Kiriyama-Tanaka T, Mizuno K. Isolation of type I collagen homotrimer from human placenta with LC-MS monitoring of the alpha1(I)/alpha2(I) chain ratio. Int J Biol Macromol 2024;255:128301. 37992935 10.1016/j.ijbiomac.2023.128301

[35] Kilkenny C , Browne WJ, Cuthill IC, Emerson M, Altman DG. Improving bioscience research reporting: the ARRIVE guidelines for reporting animal research. PLoS Biol 2010;8:e1000412. 20613859 10.1371/journal.pbio.1000412 PMC2893951

[36] Wang X , Dai W, Gao C, Zhang L, Wan Z, Zhang T, Wang Y, Tang Y, Yu Y, Yang X, Cai Q. Spatiotemporal modulated scaffold for endogenous bone regeneration via harnessing sequentially released guiding signals. ACS Appl Mater Interfaces 2023;15:58873–87. 38058149 10.1021/acsami.3c13963

[37] Yang B , Li T, Wang Z, Zhu Y, Niu K, Hu S, Lin Z, Zheng X, Jin X, Shen C. Ruxolitinib-based senomorphic therapy mitigates cardiomyocyte senescence in septic cardiomyopathy by inhibiting the JAK2/STAT3 signaling pathway. Int J Biol Sci 2024;20:4314–40. 39247818 10.7150/ijbs.96489 PMC11379065

[38] Sung H-W , Huang R-N, Huang LLH, Tsai C-C. In vitro evaluation of cytotoxicity of a naturally occurring cross-linking reagent for biological tissue fixation. J Biomater Sci Polym Ed 1999;10:63–78. 10091923 10.1163/156856299x00289

[39] Fessel G , Cadby J, Wunderli S, van Weeren R, Snedeker JG. Dose- and time-dependent effects of genipin crosslinking on cell viability and tissue mechanics—toward clinical application for tendon repair. Acta Biomater 2014;10:1897–906. 24384123 10.1016/j.actbio.2013.12.048

[40] Wildemann B , Ignatius A, Leung F, Taitsman LA, Smith RM, Pesantez R, Stoddart MJ, Richards RG, Jupiter JB. Non-union bone fractures. Nat Rev Dis Primers 2021;7:57. 34354083 10.1038/s41572-021-00289-8

[41] Wang Y , Wang Y, Wang X, Li X, Yu Y, Kaplan DL, Cai Q. Biodegradable and electroactive cryogel microspheres for neurovascularized bone regeneration. Matter 2025;8:102366.

[42] Wu M , Liu H, Zhu Y, Chen F, Chen Z, Guo L, Wu P, Li G, Zhang C, Wei R, Cai L. Mild photothermal‐stimulation based on injectable and photocurable hydrogels orchestrates immunomodulation and osteogenesis for high‐performance bone regeneration. Small 2023;19:e2300111. 37191242 10.1002/smll.202300111

[43] Liu J , Zhao Y, Zhang Y, Yao X, Hang R. Exosomes derived from macrophages upon Zn ion stimulation promote osteoblast and endothelial cell functions. J Mater Chem B 2021;9:3800–7. 33899897 10.1039/d1tb00112d

[44] Lu T , Wang J, Yuan X, Tang C, Wang X, He F, Ye J. Zinc-doped calcium silicate additive accelerates early angiogenesis and bone regeneration of calcium phosphate cement by double bioactive ions stimulation and immunoregulation. Biomater Adv 2022;141:213120. 36122428 10.1016/j.bioadv.2022.213120

[45] Bonaventura P , Benedetti G, Albarede F, Miossec P. Zinc and its role in immunity and inflammation. Autoimmun Rev 2015;14:277–85. 25462582 10.1016/j.autrev.2014.11.008

[46] Gammoh NZ , Rink L. Zinc in infection and inflammation. Nutrients 2017;9:1–25. 10.3390/nu9060624 PMC5490603 28629136

[47] Banerjee S , Biehl A, Gadina M, Hasni S, Schwartz DM. JAK-STAT signaling as a target for inflammatory and autoimmune diseases: current and future prospects. Drugs 2017;77:521–46. 28255960 10.1007/s40265-017-0701-9 PMC7102286

[48] Basquiera AL , Soria NW, Ryser R, Salguero M, Moiraghi B, Sackmann F, Sturich AG, Borello A, Berretta A, Bonafe M, Barral JM, Palazzo ED, Garcia JJ. Clinical significance of V617F mutation of the JAK2 gene in patients with chronic myeloproliferative disorders. Hematology 2009;14:323–30. 19941738 10.1179/102453309X12473408860226

[49] Jia B , Yang H, Zhang Z, Qu X, Jia X, Wu Q, Han Y, Zheng Y, Dai K. Biodegradable Zn-Sr alloy for bone regeneration in rat femoral condyle defect model: in vitro and in vivo studies. Bioact Mater 2021;6:1588–604. 33294736 10.1016/j.bioactmat.2020.11.007 PMC7691683

[50] Kambe T , Tsuji T, Hashimoto A, Itsumura N. The physiological, biochemical, and molecular roles of zinc transporters in zinc homeostasis and metabolism. Physiol Rev 2015;95:749–84. 26084690 10.1152/physrev.00035.2014

[51] Liu W , Li J, Cheng M, Wang Q, Yeung KWK, Chu PK, Zhang X. Zinc-Modified sulfonated polyetheretherketone surface with immunomodulatory function for guiding cell fate and bone regeneration. Adv Sci (Weinh) 2018;5:1800749. 30356934 10.1002/advs.201800749 PMC6193167

[52] Wang T , Bai J, Lu M, Huang C, Geng D, Chen G, Wang L, Qi J, Cui W, Deng L. Engineering immunomodulatory and osteoinductive implant surfaces via mussel adhesion-mediated ion coordination and molecular clicking. Nat Commun 2022;13:160. 35013289 10.1038/s41467-021-27816-1 PMC8748715

[53] Hrabák A , Bögel G, Murányi J, Tamási V, Németh K, Szokol B, Kukor Z, Kardon T, Őrfi L. Decreasing effects of protein kinase inhibitors on the expression of NOS2 and inflammatory cytokines and on phagocytosis in rat peritoneal macrophages is partly related to repolarization. Mol Immunol 2023;153:10–24. 36402067 10.1016/j.molimm.2022.11.002

[54] Lawrence T , Natoli G. Transcriptional regulation of macrophage polarization: enabling diversity with identity. Nat Rev Immunol 2011;11:750–61. 22025054 10.1038/nri3088

[55] Tugal D , Liao X, Jain MK. Transcriptional control of macrophage polarization. Arterioscler Thromb Vasc Biol 2013;33:1135–44. 23640482 10.1161/ATVBAHA.113.301453

