# Supplementary material for: Zinc silicate modulates bone substitute degradation following macrophage activation via the JAK/STAT pathway and expedites the initiation of bone repair: in vitro and in vivo studies
Source: Regen Biomater. 2026 Mar 5;13:rbag037. doi: 10.1093/rb/rbag037 (PMC13223734; doi:10.1093/rb/rbag037)
Supplement: rbag037_Supplementary_Data [file rbag037_supplementary_data.zip › Supplement data_revised.docx]

| **Table S1** Collagen protein components extracted from pig skin identified via LC-MS (top 15) | | | |
| --- | --- | --- | --- |
|  | Accession | -10lgP | Description |
| 1 | A0A5G2QQE9\|A0A5G2QQE9_PIG | 208.12 | Collagen type I alpha 1 chain |
| 2 | A0A8D0MXF3\|A0A8D0MXF3_PIG | 208.12 | Collagen type I alpha 1 chain |
| 3 | A0A8D1NJM6\|A0A8D1NJM6_PIG | 207.74 | Collagen alpha-1(I) chain |
| 4 | A0A287A1S6\|A0A287A1S6_PIG | 207.74 | Collagen alpha-1(I) chain |
| 5 | A0A1S7J210\|A0A1S7J210_PIG | 207.74 | Alpha1 chain of type I collagen |
| 6 | A0A4X1USL6\|A0A4X1USL6_PIG | 207.63 | Collagen alpha-1(I) chain |
| 7 | A0A8D1Z2N0\|A0A8D1Z2N0_PIG | 207.31 | Collagen type I alpha 1 chain |
| 8 | A0A4X1USK6\|A0A4X1USK6_PIG | 207.25 | Collagen alpha-1(I) chain |
| 9 | A0A8D2C974\|A0A8D2C974_PIG | 206.59 | Collagen type I alpha 1 chain |
| 10 | A0A8D0J309\|A0A8D0J309_PIG | 203.70 | Collagen alpha-1(I) chain |
| 11 | A0A8D0IQS0\|A0A8D0IQS0_PIG | 203.30 | Collagen alpha-1(I) chain |
| 12 | A0A8D0LLK6\|A0A8D0LLK6_PIG | 203.30 | Collagen alpha-1(I) chain |
| 13 | A0A287BLD2\|A0A287BLD2_PIG | 196.01 | Collagen type I alpha 1 chain |
| 14 | A0A1S7J1Y9\|A0A1S7J1Y9_PIG | 176.93 | Alpha2 chain of type I collagen |
| 15 | A0A4X1U043\|A0A4X1U043_PIG | 176.93 | Fibrillar collagen NC1 domain-containing protein |

| **Table S2.** Gene expression levels of M1/M2 macrophage markers in each group on day 3 | | | | |
| --- | --- | --- | --- | --- |
|  | 0ZCH | 5ZCH | 10ZCH | 15ZCH |
| iNOS gene expression level | 0.40±0.10 | 0.67±0.15 | 1.20±0.10 | 1.37±0.15 |
| CD86 gene expression level | 0.53±0.15 | 0.9±0.26 | 1.4±0.1 | 1.53±0.15 |
| Arg1 gene expression level | 0.43±0.12 | 0.63±0.15 | 0.53±0.15 | 0.40±0.10 |
| CD206 gene expression level | 0.50±0.20 | 0.57±0.25 | 0.90±0.20 | 0.70±0.20 |

| **Table S3.** Gene expression levels of M1/M2 macrophage markers in each group on day 7 (x̄±SD) | | | | |
| --- | --- | --- | --- | --- |
|  | 0ZCH | 5ZCH | 10ZCH | 15ZCH |
| iNOS gene expression level | 0.60±0.10 | 1.13±0.21 | 1.77±0.15 | 2.30±0.20 |
| CD86 gene expression level | 0.80±0.26 | 1.37±0.25 | 1.97±0.21 | 2.17±0.15 |
| Arg1 gene expression level | 0.60±0.10 | 0.67±0.12 | 0.43±0.06 | 0.50±0.10 |
| CD206 gene expression level | 0.67±0.15 | 0.83±0.31 | 1.37±0.21 | 1.30±0.10 |

| **Table S4.** Residual rate of the composites after coculture with macrophages (x̄±SD) | | | | |
| --- | --- | --- | --- | --- |
|  | 0ZCH | 5ZCH | 10ZCH | 15ZCH |
| 0d | 100±0 | 100±0 | 100±0 | 100±0 |
| 1d | 96.33±1.53 | 94.00±1.00 | 93.67±1.53 | 93.00±2.00 |
| 3d | 94.33±0.58 | 91.67±1.53 | 88.33±2.08 | 86.00±2.00 |
| 7d | 91.67±1.53 | 85.00±2.00 | 83.00±1.00 | 81.33±1.53 |
| 14d | 88.67±1.53 | 0.00±2.00 | 74.67±2.52 | 71.33±1.53 |
| 21d | 85.33±1.53 | 72.00±2.00 | 57.67±4.51 | 44.67±3.79 |
| 28d | 78.33±2.08 | 44.00±4.00 | 27.00±3.61 | 17.33±2.52 |
| total | 90.67±7.01 | 80.95±17.88 | 74.90±24.04 | 70.52±28.07 |

| **Table S5.** Absorbance values of the media at different times (x̄±SD) | | | | |
| --- | --- | --- | --- | --- |
|  | 0ZCH | 5ZCH | 10ZCH | 15ZCH |
| 7d | 1.30±0.20 | 1.53±0.15 | 1.50±0.10 | 1.63±0.15 |
| 14d | 1.23±0.15 | 1.57±0.15 | 1.60±0.20 | 1.73±0.15 |
| 21d | 1.23±0.15 | 1.63±0.15 | 1.80±0.20 | 2.07±0.21 |
| total | 1.26±0.15 | 1.58±0.14 | 1.63±0.20 | 1.81±0.25 |

| **Table S6.** Haematological analysis (x̄±SD) | | | | | |  |
| --- | --- | --- | --- | --- | --- | --- |
|  | Control (n=3) | 0ZCH (n=3) | 5ZCH (n=3) | 10ZCH (n=3) | 15ZCH |  |
| White blood cell(*10^9^/L) | 1.47±0.36 | 5.30±0.15 | 2.27±0.11 | 3.39±0.29 | 4.46±0.27 |  |
| Neu% | 9.87±2.44 | 8.37±0.83 | 13.30±1.35 | 13.53±0.45 | 4.83±0.06 |  |
| Lym% | 80.0±2.92 | 79.67±0.55 | 67.77±1.56 | 69.40±1.95 | 83.97±1.53 |  |
| Eosinophilic counts(*10^9^/L) | 0.03±0.02 | 0.04±0.02 | 0.02±0.01 | 0.04±0.01 | 0.07±0.02 |  |
| Platelet count(*10^9^/L) | 547.33±188.32 | 594.0±19.0 | 732.67±19.09 | 1010.33±37.50 | 1063.67±91.27 |  |
| Mean platelet volume(fl) | 7.00±0.35 | 7.00±0.0 | 7.20±0.20 | 7.63±0.12 | 7.77±0.12 |  |
| Thrombocytocrit(*100%) | 0.38±0.12 | 0.42±0.01 | 0.53±0.02 | 0.77±0.02 | 0.82±0.06 |  |
| Platelet distribution width(fl) | 16.83±0.31 | 15.83±0.06 | 16.27±0.12 | 16.07±0.06 | 16.07±0.21 |  |
| Erythrocyte count(*10^12^/L) | 5.46±0.90 | 5.81±0.11 | 5.90±0.24 | 4.92±0.09 | 5.44±0.52 |  |
| Hemoglobin(g/L) | 112.33±17.39 | 118.67±2.89 | 117.33±3.79 | 100.0±2.0 | 115.67±7.51 |  |
| Hematocrit(%) | 35.47±6.12 | 38.87±0.78 | 40.30±1.91 | 33.03±0.68 | 36.97±3.45 |  |
| Alanine aminotransferase(U/L) | 55.80±10.06 | 54.13±7.20 | 47.48±12.47 | 43.33±0.87 | 43.37±15.04 |  |
| Aspartate aminotransferase(U/L) | 216.74±44.69 | 236.11±32.26 | 196.50±29.60 | 177.40±8.97 | 216.56±25.74 |  |
| Alkaline phosphatase(U/L) | 173.84±18.16 | 224.35±12.69 | 254.39±4.63 | 348.37±91.90 | 227.63±125.73 |  |
| Blood urea nitrogen(mmol/L) | 11.23±1.49 | 11.71±1.85 | 15.50±1.74 | 10.46±0.30 | 13.34±0.83 |  |
| Creatinine(μmol/L) | 33.14±4.34 | 40.71±9.88 | 46.50±2.81 | 38.74±0.44 | 45.80±10.23 |  |
| Creatine kinase isoenzyme(ng/mL) | 472.10±320.90 | 560.55±312.64 | 741.36±87.47 | 744.34±32.53 | 661.16±346.22 |  |
| Lactate dehydrogenase(U/L) | 1590.95±719.30 | 2562.92±906.05 | 1918.38±598.61 | 1995.93±292.72 | 2446.97±550.12 |  |
| **Note:**  Neu% the percentage of neutrophils; Lym%: the percentage of lymphocyte. | | | | | |  |
|  |  |  |  |  |  |  |

| **Table S7.** Gene expression levels of M0/M1/M2 macrophages in tissues as determined via qRT‒PCR (x̄±SD) | | | | | |
| --- | --- | --- | --- | --- | --- |
|  | control | 0ZCH | 5ZCH | 10ZCH | 15ZCH |
| F4/80 gene expression level | 1.00±0.38 | 0.94±0.22 | 0.87±0.05 | 1.30±0.46 | 1.22±0.14 |
| CD68 gene expression level | 1.00±0.18 | 1.11±0.34 | 1.20±0.34 | 1.54±0.28 | 1.24±0.32 |
| iNOS gene expression level | 1.00±0.22 | 1.88±0.61 | 5.96±0.94 | 8.34±0.94 | 6.34±0.78 |
| CD86 gene expression level | 0.20±0.07 | 0.29±0.10 | 0.77±0.38 | 1.42±0.28 | 1.00±0.40 |
| Arg1 gene expression level | 1.00±0.61 | 7.39±2.22 | 9.86±2.08 | 14.10±2.86 | 11.40±2.93 |
| CD206 gene expression level | 1.00±0.04 | 2.06±0.33 | 2.38±0.52 | 2.01±0.48 | 1.02±0.06 |

| **Table S8.** Degradation volume of the different composites at different times (x̄±SD, n=3,Unit: mm^3^) | | | | |
| --- | --- | --- | --- | --- |
|  | 0ZCH | 5ZCH | 10ZCH | 15ZCH |
| 4 weeks | 1.56±0.08 | 3.11±0.38 | 5.75±0.41 | 4.03±0.56 |
| 8 weeks | 3.19±0.19 | 5.91±0.75 | 9.34±0.39 | 8.55±0.46 |
| 12 weeks | 5.74±0.37 | 9.44±0.61 | 12.52±0.71 | 11.32±0.34 |

| **Table S9.** Gene expression levels of macrophages of different phenotypes determined by qRT‒PCR after the administration of ruxolitinib (x̄±SD) | | | |
| --- | --- | --- | --- |
|  | 0ZCH | 10ZCH | 10ZCH+Ru |
| F4/80 gene expression level | 0.90±0.08 | 0.91±0.02 | 0.79±0.07 |
| iNOS gene expression level | 1.00±0.04 | 1.63±0.13 | 1.00±0.09 |
| CD86 gene expression level | 1.00±0.25 | 2.40±0.28 | 1.41±0.17 |
| Arg1 gene expression level | 1.00±0.12 | 2.90±0.12 | 0.92±0.02 |
| CD206 gene expression level | 1.00±0.26 | 2.99±0.27 | 1.48±0.16 |

Note Ru: ruxolitinib.
