# Supplementary material for: Zinc silicate modulates bone substitute degradation following macrophage activation via the JAK/STAT pathway and expedites the initiation of bone repair: in vitro and in vivo studies
Source: Regen Biomater. 2026 Mar 5;13:rbag037. doi: 10.1093/rb/rbag037 (PMC13223734; doi:10.1093/rb/rbag037)
Supplement: rbag037_Supplementary_Data [file rbag037_supplementary_data.zip › Title page.docx]

**Zinc Silicate Modulates Bone Substitute Degradation Following Macrophage Activation via the JAK/STAT Pathway and Expedites** **the Initiation of Bone Repair: In Vitro and In Vivo Studies**

**Authors**

**Author name:** *Jingdi Chen ^1,2 #^, Xiaotian Hao ^1 #^, Chunxing Xian ^1 #^, Xiang He ^1^, Taoran Wang ^1^, Jiakai Gao^1^, Tao Liu ^2*^, Wei Wu ^3 *^ Zhao Yang ^4,1*^ and* *Long Bi ^1*^*.

#. These authors contributed equally to this work.

*. The corresponding authors is Dr. *Long Bi****,*** *Zhao Yang****,*** Wei Wu and Tao Liu.

**Final Degrees:**

Jingdi Chen: Master

Xiaotian Hao: Master

Chunxing Xian: Master

Xiang He: Master

Taoran Wang: Master

Jiakai Gao: Master

Tao Liu: M.D.

Wei Wu: M.D.

Zhao Yang: M.D.

Long Bi: M.D.

**Author affiliations:**

1 Department of Orthopedics, Xijing Hospital, Air Force Medical University, Xi'an, 710032, China

2 Department of Orthopedics, 95829 Military Hospital in PLA, Wuhan, 430000, China

3 Department of Critical Care Medicine, Renmin Hospital of Wuhan University, Wuhan, 430000, China

4 Ankle Trauma and Degeneration Ward, Honghui Hospital, Xi’an Jiaotong University, Xi’an, Shaanxi 710054, P.R. China.

**Email Address:**

Jingdi Chen, E-mail: chenjd2012@163.com

Xiaotian Hao, E-mail: 1936319928@qq.com

Chunxing Xian, E-mail: 1739333744@qq.com

Xiang He, E-mail: caruya@qq.com

Taoran Wang, E-mail: 723801134@qq.com

Jiakai Gao, E-mail: gaojiakai@aliyun.com

Tao Liu, E-mail: liut202108@163.com

Wei Wu, E-mail: weiwu2012@whu.edu.cn

Zhao Yang, E-mail: [yangzhaofirst@126.com](mailto:yangzhaofirst@126.com)

Long Bi, E-mail: [bilong@fmmu.edu.cn](mailto:bilong@fmmu.edu.cn)

Other information is as following:

Tel: +86-29-84771014

Fax: +86-29-84771014

**Funding：**This work was supported by National Key Research and Development Program of China(2023YFC2509905), the Joint Founding Project of Innovation Research Institute, Xijing hospital (LHJJ24KQ05), the National Natural Science Foundation of China (82302418).We thank Professor Qingfeng Zeng (general manager of Xi 'an Dianyun Biotechnology Co., Ltd.) for providing considerable help in the preparation of the 3D-printed biomaterials.

**Ethical Review Committee Statement:** Each author certifies that his institution approved the human protocol for this investigation and that all investigations were conducted in conformity with ethical principles of research.
