# Supplementary material for: Zinc silicate modulates bone substitute degradation following macrophage activation via the JAK/STAT pathway and expedites the initiation of bone repair: in vitro and in vivo studies
Source: Regen Biomater. 2026 Mar 5;13:rbag037. doi: 10.1093/rb/rbag037 (PMC13223734; doi:10.1093/rb/rbag037)
Supplement: rbag037_Supplementary_Data [file rbag037_supplementary_data.zip › Supplement figure.docx]

**Figure S1**


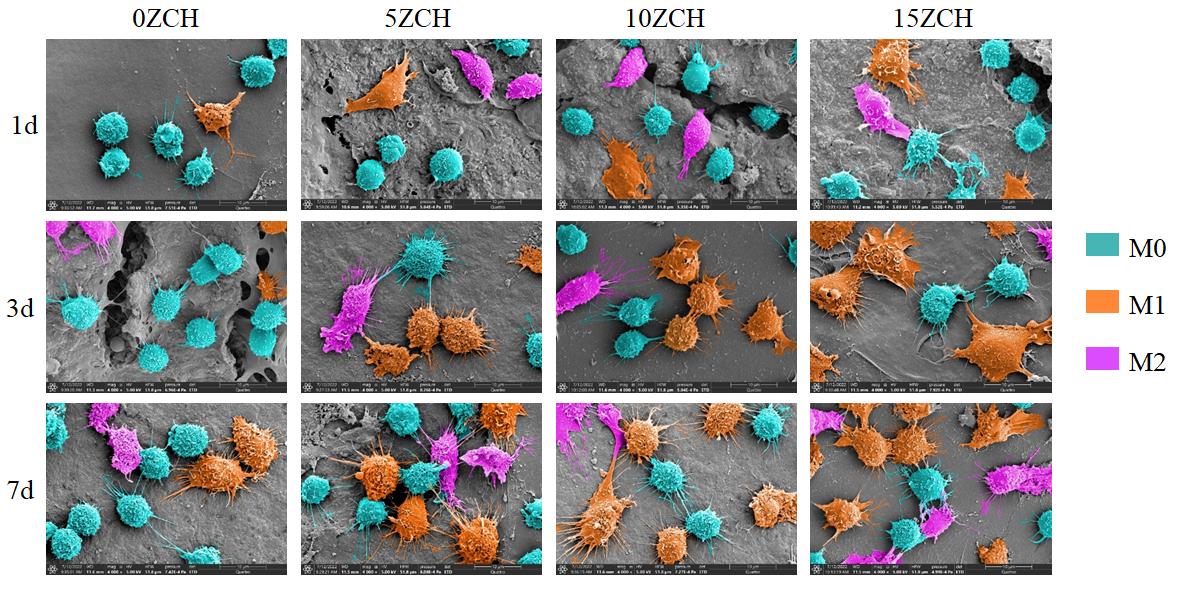


**Figure S1.** Scanning electron microscopy of the effects of zinc ion-containing silicic acid zinc composites of different concentrations on macrophage polarization. Based on cellular morphological features, macrophages were automatically identified and classified into three subtypes: M0 (light blue), M1 (orange), and M2 (purple).

**Figure S2**


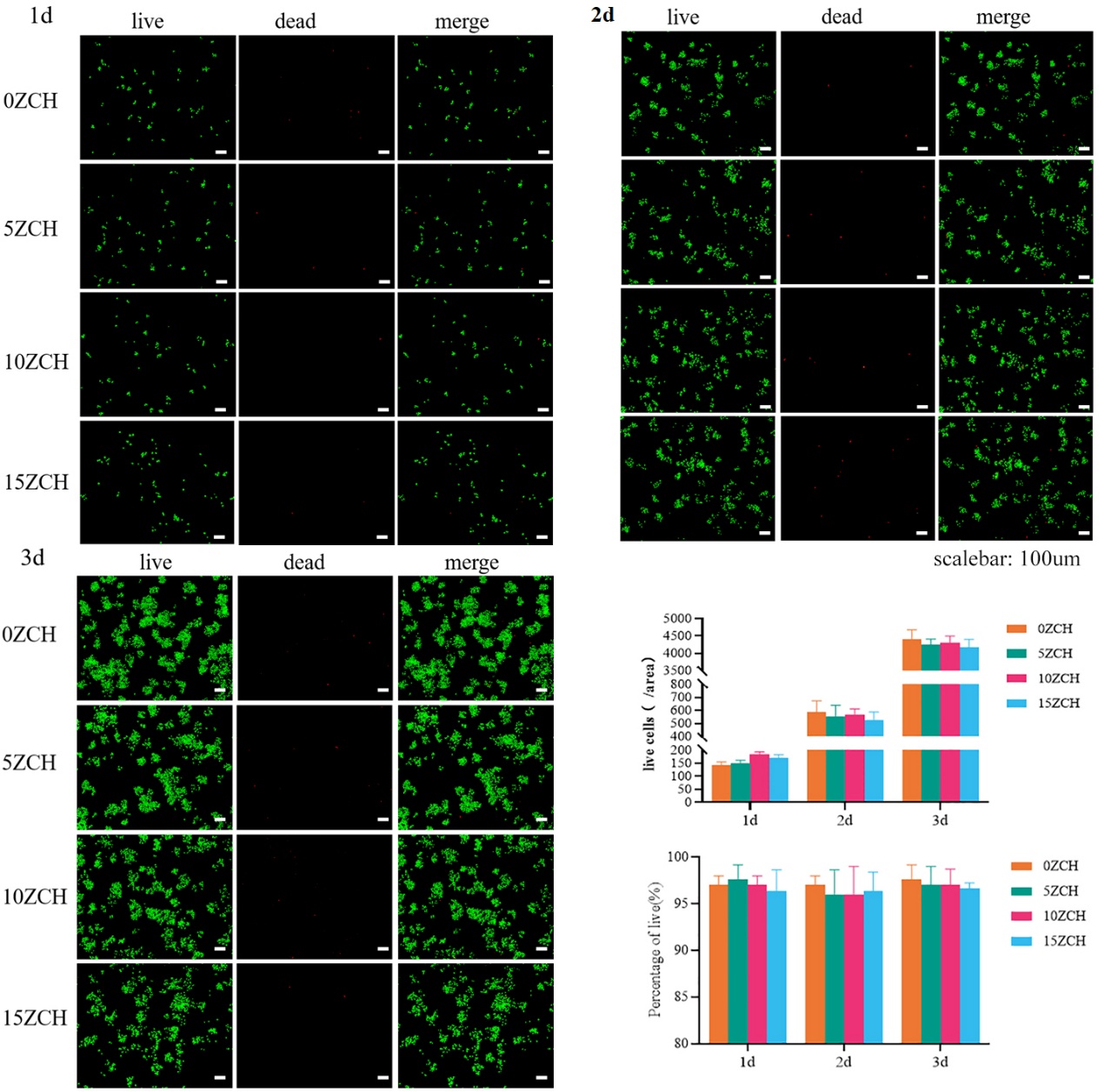


**Figure S2.** Macrophages were co-cultured with composite scaffold extracts for 1, 2, and 3 days, followed by a live/dead staining assay to assess cell viability (red: dead cells; green: live cells). As shown, the bar chart illustrates the number and proportion of live cells at each time point.

**Figure S3**


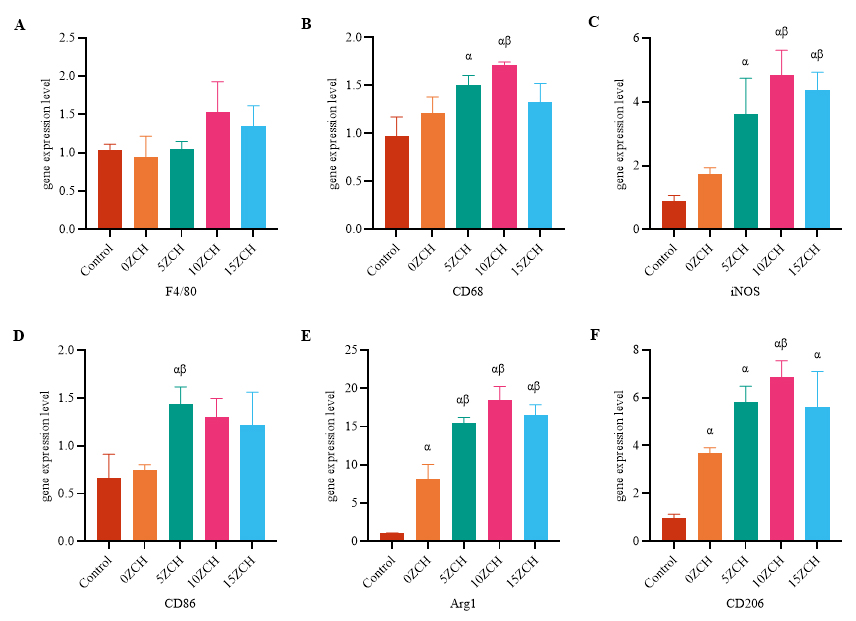


**Figure S3**. Investigation of the changes in macrophage phenotype caused by the composites in vivo. Panels A-F show the gene expression levels of M0 macrophages (Panels A-B), M1 macrophages (Panels C-D), and M2 macrophages (Panels E-F) in tissues as determined via qRT‒PCR four weeks after surgery. The data are presented as the mean± SD (n=3). α represents P<0.05 compared with the control group, β represents P<0.05 compared with the 0ZCH group.
